# Association of healthy lifestyle with life expectancy free of five major disabilities in Chinese older adults

**DOI:** 10.7189/jogh.14.04034

**Published:** 2024-01-12

**Authors:** Yao Li, Yuhong Tang, Yijun Xie, Hui Liu, Hengjing Wu

**Affiliations:** 1 Clinical Center for Intelligent Rehabilitation Research, Shanghai YangZhi Rehabilitation Hospital (Shanghai Sunshine Rehabilitation Center), School of Medicine, Tongji University, Shanghai, China; 2 School of Medicine, Tongji University, Shanghai, China

## Abstract

**Background:**

Whether and to what extent multiple healthy lifestyles affect the longevity of people with disabilities, including those in basic activities of daily living, mobility, vision, hearing and cognition, is crucial to policymakers. We aimed to determine the impact of combined lifestyles on life expectancy (LE) lived with and without five disabilities.

**Methods:**

We recruited participants (n = 15 121 from the China Longitudinal Healthy Longevity Survey between 2008 and 2018. Healthy lifestyle levels were estimated from six factors: smoking, drinking, physical exercise, diet, cognitive activity, and sleep, which we categorised as favourable and unfavourable using the latent class growth mixture model throughout the follow-up period. We used Multi-state Markov models to assess the different disability stages of LE.

**Results:**

Of the total LE at age 65, older adults with a favourable lifestyle spent 59.60% (disability-free LE (DFLE) = 10.24 years) without five disabilities in combination, whereas those with unfavourable lifestyle spent 56.74% (DFLE = 7.28 years). Furthermore, the percentage of DFLE was 64.98 (7.71 years) and 68.38 (9.91 years) in males with unfavourable and favourable lifestyle levels, respectively, and 47.92 (6.62 years) and 55.12 (10.30 years) for females. Compared to older adults with low socioeconomic status (SES) and unfavourable lifestyle level, those with lower SES and favourable lifestyle level had more 3.77 years of DFLE, those with higher SES and unfavourable lifestyle level had more 1.94 years, as well as those with higher SES and favourable lifestyle level had more 5.10 years at age 65. Corresponding associations were found separately for each of the five individual disabilities.

**Conclusions:**

A favourable lifestyle level was associated with longer total LE along with a higher proportion of DFLE and may contribute to narrowing socioeconomic health inequalities. Policymakers should develop lifestyle interventions and scale up rehabilitation services in primary care, thereby delaying disabilities to later ages, especially in low- and middle-income countries.

The World Health Organization (WHO) has defined healthy ageing as a process of maintaining the functional ability to enable well-being in older age, and health life expectancy (HALE) is a crucial dimension of health ageing [1]. Based on United Nations (UN) estimates (World Population Prospects 2022) [2], the world’s population aged 65 years and over is set to increase from 780 million in 2022 to 1.6 billion in 2050, and the global life expectancy (LE) has extended by about 8.8 years over the past 30 years. Relevant studies have shown that estimated disability-free LE (DFLE) at age 65 ranged from 11.5–18.0 years for males and 11.7–17.0 years for females in different countries [3–6], but the increase in DFLE was smaller than the increase in overall LE during this period, indicating more years lived in disability or functional decline, which has a significant impact on the financial costs and health care burden for individuals and the nation [7,8]. Furthermore, two-thirds of older persons live in low- and middle-income countries (LMICs), and by 2050, this proportion will reach 80% [9]. However, the situation in LMICs has been much less extensively studied, with limited data available. Therefore, identifying modifiable risk factors that increase the proportion of DFLE and the populations most susceptible to these factors is critical to laying the foundation for laying the groundwork for long-term integrated care for older adults in LMICs.

Previous studies have suggested that combining multiple lifestyle factors may prolong total LE and relate to the LE free from major chronic diseases [10–13]. Despite some findings implicating that single lifestyle factors (including smoking, alcohol, physical exercise, diet, or sleep) might affect both total LE and DFLE [14–16], how healthy lifestyles in combination affect LE free from multiple types of disability is poorly understood. In addition, some studies have shown that lower SES was associated with shorter total LE and DFLE [14,17,18]. Still, it is unclear whether lifestyles and socioeconomic status (SES) jointly affect DFLE. Moreover, much research in DFLE has focused on basic activities of daily living (BADL) [4,6,16–19], and other disability types of LE have not been exhaustively studied. Preventing premature mortality and prolonging the proportion of DFLE are major public health challenges and policy imperatives.

Given the positive association between a combined healthy lifestyle and HALE and the opposite association between lower SES and DFLE, it is plausible to hypothesise that adherence to healthy lifestyles may independently prolong the proportion of LE free from the significant types of disability and attenuate the loss of disability-free years associated with lower SES. Thus, we aimed to explore the associations between multiple healthy lifestyles with LE free of five major disabilities, including the disabilities in BADL, mobility, vision, hearing and cognition. We also investigated the joint associations of lifestyles and SES with DFLE.

## METHODS

### Study design and participants

This study was based on the China Longitudinal Healthy Longevity Survey (CLHLS), an ongoing dynamic cohort study conducted among older adults randomly selected from 22 provinces in China. A more detailed description of the sampling design can be found elsewhere [20]. The survey was initiated in 1998 with follow-up every two to three years, approved by the Biomedical Ethics Committee of Peking University (IRB00001052-13,074), and all participants or their respondents provided written informed consent.

The multi-state model requires participants to respond to at least two waves of surveys to examine transitions between the states. The final analysis included 15 121 participants with baseline measures collected between 2008–14, and data have since been linked to mortality records. The exclusion criteria were the following: under 65 years old; responded to only one wave survey after excluding missing date at death; responded to only one wave survey after excluding missing states and lifestyles; and had systematic registration error (Figure S1 in the **Online Supplementary Document**).

### Assessment of healthy lifestyle

Healthy lifestyle status was assessed by six lifestyle factors: smoking, drinking, physical exercise, diet, cognitive activity, and sleep [21,22]. For smoking, participants were categorised as current smokers, former smokers, and never smokers, with never smoking being considered a healthy lifestyle factor. We used a similar evaluation to define drinkers and physical exercisers. For diet, the frequency of 13 food items consumed by participants was recorded (fruits, vegetables, fish, meat, dairy products, vegetable oil, eggs, whole grain, legumes, nuts, tea, garlic, mushrooms, and algae), with at least seven foods per week considered healthy. We considered participation at least once weekly to be healthy for cognitive activities (writing, reading, playing cards, mahjong, watching television, and listening to the radio). We define healthy sleep as time ≥7 and ≤9 hours and very good/good/fair quality. Ultimately, we identified two distinct trajectories according to healthy lifestyle factors over the 10 years by the latent class growth mixture model (LCGMM) (favourable and unfavourable group) (Figure S2 in the **Online Supplementary Document**).

### Assessments of five major disabilities and death

BADL were measured with the six aspects in CLHLS: bathing, dressing, toileting, indoor moving, continence of defecation, and eating. Each item was asked with three answers (complete independence, partial dependence, and complete dependence). BADL disability was defined as one or more of the six aspects that cannot be completely independence [23]. The mobility function was assessed by asking participants, ‘Can you walk continuously for 1 km at a time by yourself?’ If the participant reported difficulty with this task (a little difficult or unable to do), it was defined as having a mobility disability [23]. Hearing function was assessed if participants could hear clearly what the interviewers said during the questionnaire survey. Four options were available: yes, without a hearing aid; yes, but needs a hearing aid; partly, despite a hearing aid; and No. If interviewers selected the third or fourth item, participants were identified as having a hearing disability [24]. Vision function was assessed by asking participants if they could see the circle on the card and distinguish the direction of the break in the circle with a flashlight shining on it. Four choices could be selected: can see and distinguish; can see but can’t distinguish; can’t see; and blind. Participants were categorised as having vision disability if they could not see the circle or were blind (the third or fourth item) [24]. The Mini-Mental State Examination (MMSE) of the Chinese version was utilised to assess participants’ cognitive function, which has been verified with good reliability and validity [25]. More details about the Chinese version of the MMSE are shown in Table S1 in the **Online Supplementary Document**. We used the MMSE scores of 18, 20, and 24 as the cut-off points for the participants without formal education, primary school education (one to six years), and secondary school or higher education (>6 years). We defined corresponding participants below these cut-off points as having cognitive disability [26].

We investigated all-cause mortality. The vital status and date of death (for participants who died by the end of the study) of the deceased participants were ascertained by the death certificates provided by their family members or by the local neighbourhood committee during the follow-up survey conducted in 2011, 2014, and 2018. The duration of follow-up was calculated as the time interval between the first interview date and the date of death.

### Assessments of covariates

Sociodemographic information and health-related indicators were considered to control the potential bias. The former covariates included age, gender (male or female), region (east, centre, or west), marital status (married or widowed, divorced/separated/single), and SES. SES was assessed by a composite of the socioeconomic vulnerability index (SEVI) with six components (education, occupation, economic independence, family economic status, timely access to health care services, and place of residence) (Table S2 in the **Online Supplementary Document**) [27]. SEVI scores range from zero to one, with higher scores indicating lower levels of SES. All participants were divided into lower and higher SES groups according to the results of the LCGMM at SEVI (Figure S2 in the **Online Supplementary Document**). We collected health-related covariates at baseline and each follow-up, including body mass index (BMI) and medical illnesses. Medical illnesses were based on self-reported hypertension, diabetes, heart disease, stroke and other cerebrovascular, and dyslipidaemia.

### Statistical analysis

Assuming a ratio of 40:60 for the size of people in the favourable vs unfavourable lifestyle group, with a *P* < 0.05, a power of 90%, an odds ratio (OR) of 0.7, and the disability/death rate of 30% in the favourable group [3,21], we calculated the sample size to be at least 720 for the favourable group and 1080 for the unfavourable group.

We applied LCGMM to model healthy lifestyle and SES longitudinal trajectories over the follow-up period in older adults and to identify distinct subgroups following similar patterns. We compared two- to five-class LCGMM models iterating 1st- to 3rd-degree fractional polynomials. The best-fitting one was determined by the minimum absolute value of the Bayesian information criterion, the average posterior probability of each subgroup not less than 0.7, the proportion of each subgroup with posterior probability greater than 0.7 not less than 0.65, and the membership of each subgroup not less than 5% of the total population (Tables S3 and S4 in the **Online Supplementary Document**). We identified missing data for covariates through multiple imputation methods (Table S5 in the **Online Supplementary Document**). We assumed data to be missing at random, with a predictive mean matching method for missing continuous variables and a logistic regression model for missing binary variables. If distributed normally by the Kolmogorov-Smirnov test, we presented continuous variables as means and standard deviations. Otherwise, medians and interquartile ranges were applied. Numbers and proportions presented categorical variables. We used descriptive statistics to summarise the baseline characteristics.

We used population-based multi-state life tables to calculate the total LE and years lived with and without the five major disabilities. We built six multi-state life tables, one with a combination of the disabilities in BADL, mobility, vision, hearing and cognition, and five with individual disabilities. To assess the association between a healthy lifestyle and LE free of five major disabilities, we took into account three states (free of disability, presence of disability, and death) and four transitions between states (from non-disability to incident disability, from non-disability to mortality, from disability to mortality, and from disability to non-disability) in a Markov multi-state transition model (Figure S3 in the **Online Supplementary Document**). First, we calculated the overall transition rates for each transition using survival analysis with Gompertz distribution. Second, we calculated hazard ratios by healthy lifestyle for each transition in Markov multi-state transition models. We adjusted the models by age, gender, region, BMI, marital status, medical illnesses (hypertension, diabetes, heart disease, stroke and other cerebrovascular, and dyslipidaemia), and SES. Third, we calculated the proportion of lifestyle levels among the sub-study population for each transition. Finally, we combined overall transition rates, hazard ratios and the proportion of lifestyle levels to derive weighted transition rates. These were used to create multi-state life tables to calculate total LE and LE with and without disabilities for each group. The multi-state life table started at age 65 and ended at age 115 years. We calculated the 95% confidence intervals (CIs) of LE estimation using bootstrapping with 1000 iterations. Considering the pathophysiological differences between genders in lifestyle, the analysis was repeatedly stratified by gender (male and female). In addition, we conducted a joint analysis of the relationship between SES and lifestyles on the DFLE, as lower SES has the opposite impact on the DFLE.

We conducted three additional sensitivity analyses to evaluate the robustness of our findings. First, we re-performed the original analysis by including only participants whose baseline wave was 2008 (n = 13 138) (sensitivity analysis 1). Second, we excluded participants who already had any of the five disabilities at baseline from the 15 121 participants (n = 11 853) and repeated the primary analysis (sensitivity analysis 2). Finally, the association of each single lifestyle factor with LE free of five major disabilities were also evaluated in models. All statistical analysis was conducted using R, version 4.2.3 (R Core Team, Vienna, Austria). Statistical significance was defined by *P <* 0.05 in two-sided testing (**Online Supplementary Document**).

## RESULTS

### The Characteristics of study participants

A total of 15 121 CLHLS participants were included in the analysis (**Table 1**). More than half (57.07%) of participants were female, with a mean age of 87.63 years (standard deviation = 11.36); 47.60% were enrolled in the favourable lifestyle group, and 40.94% were in the higher SES group. Compared to the unfavourable group, the participants were more likely to be younger, female, married, have higher SES, have more chronic diseases, and have fewer disabilities (Tables S6 and S7 in the **Online Supplementary Document**).

**Table 1:** Baseline characteristics according to two levels of healthy lifestyle*

Characteristics	Total	Favourable group	Unfavourable group	*P*-value
Age in years, mean (SD)	87.63 (11.36)	84.51 (11.40)	90.46 (10.55)	<0.001
Gender				0.003
*Male*	6492 (42.93)	3181 (44.20)	3311 (41.78)	
*Female*	8629 (57.07)	4016 (55.80)	4613 (58.22)	
Region				<0.001
*East*	7163 (47.37)	3825 (53.15)	3338 (42.13)	
*Centre*	4087 (27.03)	1786 (24.82)	2301 (29.04)	
*West*	3871 (25.60)	1586 (22.04)	2285 (28.84)	
BMI (kg/m2)				<0.001
*<18.5*	5033 (33.28)	1899 (26.39)	3134 (39.55)	
*18.5 and <24*	7926 (52.42)	3909 (54.31)	4017 (50.69)	
*≥24 and <28*	1723 (11.39)	1111 (15.44)	612 (7.72)	
*≥28*	439 (2.90)	278 (3.86)	161 (2.03)	
Marital status				<0.001
*Married*	4733 (31.30)	2883 (40.06)	1850 (23.35)	
*Widowed, divorced, separated, or single*	10 388 (68.70)	4314 (59.94)	6074 (76.65)	
Medical illnesses				
*Hypertension*	3118 (20.62)	1684 (23.40)	1434 (18.10)	<0.001
*Diabetes*	353 (2.33)	241 (3.35)	112 (1.41)	<0.001
*Heart disease*	1311 (8.67)	780 (10.84)	531 (6.70)	<0.001
*Stroke, cerebrovascular disease*	919 (6.08)	457 (6.35)	462 (5.83)	0.193
*Dyslipidaemia*	209 (1.38)	134 (1.86)	75 (0.95)	<0.001
Healthy lifestyle factors				
*Healthy diet*	7519 (49.73)	4963 (68.96)	2556 (32.26)	<0.001
*Never smoking*	12 467 (82.45)	6397 (88.88)	6070 (76.60)	<0.001
*Regular physical exercise*	3881 (25.67)	2920 (40.57)	961 (12.13)	<0.001
*Never drinking*	12 484 (82.56)	6384 (88.70)	6100 (76.98)	<0.001
*Active cognitive activity*	8848 (58.51)	5925 (82.33)	2923 (36.89)	<0.001
*Healthy sleep*	6653 (44.00)	4318 (60.00)	2335 (29.47)	<0.001
The number of healthy lifestyle factors, mean (SD)	3.43 (1.21)	4.29 (0.91)	2.64 (0.87)	<0.001
SES group				<0.001
*Lower*	8931 (59.06)	3334 (46.32)	5597 (70.63)	
*Higher*	6190 (40.94)	3863 (53.68)	2327 (29.37)	
Years of education				<0.001
*<1*	9663 (63.90)	3996 (55.52)	5667 (71.52)	
*1–6*	4104 (27.14)	2248 (31.24)	1856 (23.42)	
*>6*	1354 (8.95)	953 (13.24)	401 (5.06)	
Occupation				<0.001
*White collar*	1034 (6.84)	774 (10.75)	260 (3.28)	
*Other types*	14 087 (93.16)	6423 (89.25)	7664 (96.72)	
Economic independence				<0.001
*One’s own*	3534 (23.37)	2335 (32.44)	1199 (15.13)	
*Others*	11 587 (76.63)	4862 (67.56)	6725 (84.87)	
Economic status				<0.001
*Very rich*	161 (1.06)	109 (1.51)	52 (0.66)	
*Rich*	1883 (12.45)	1156 (16.06)	727 (9.17)	
*General*	10 410 (68.84)	5120 (71.14)	5290 (66.76)	
*Poor*	2171 (14.36)	693 (9.63)	1478 (18.65)	
*Very poor*	496 (3.28)	119 (1.65)	377 (4.76)	
Timely access to health care services				<0.001
*Yes*	14 024 (92.75)	6924 (96.21)	7100 (89.60)	
*No*	1097 (7.25)	273 (3.79)	824 (10.40)	
Place of residence				<0.001
*Urban*	2215 (14.65)	1539 (21.38)	676 (8.53)	
*Town*	2973 (19.66)	1547 (21.50)	1426 (18.00)	
*Rural*	9933 (65.69)	4111 (57.12)	5822 (73.47)	
SEVI, mean (SD)	0.64 (0.18)	0.58 (0.19)	0.69 (0.14)	<0.001
Disability				
*BADL*	3268 (21.61)	1020 (14.17)	2248 (28.37)	<0.001
*Mobility*	7577 (50.11)	2688 (37.35)	4889 (61.70)	<0.001
*Vision*	3279 (21.69)	991 (13.77)	2288 (28.87)	<0.001
*Hearing*	4046 (26.76)	1194 (16.59)	2852 (35.99)	<0.001
*Cognition*	4462 (29.51)	1247 (17.33)	3215 (40.57)	<0.001

BADL – basic activity of daily living, BMI – body mass index, SD – standard deviation, SES – socioeconomic status, SEVI – socioeconomic vulnerability index

*Presented as n (%) unless specified otherwise.

### Life expectancy and years lived with and without five disabilities in combination

**Figure 1:**
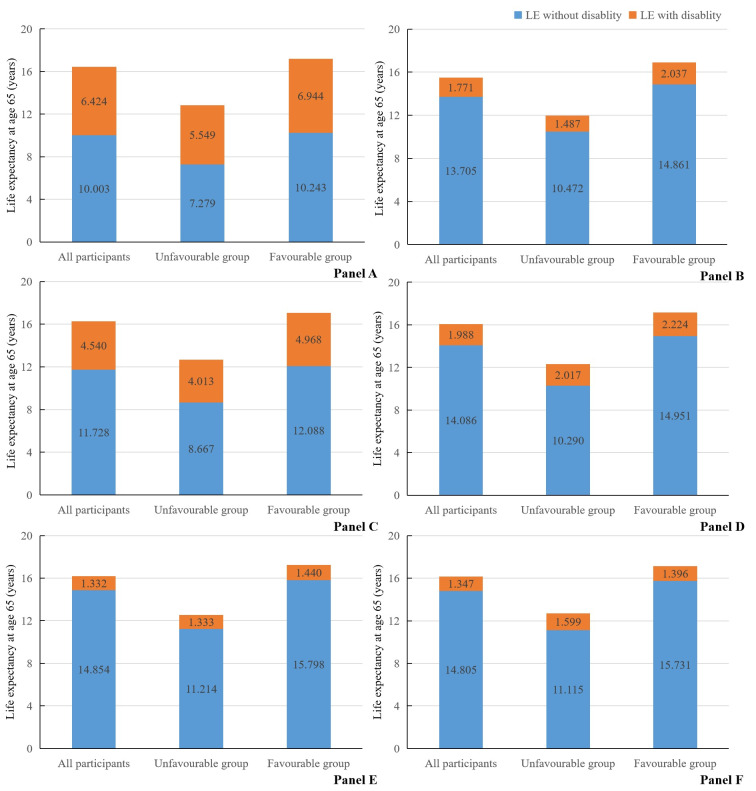
Estimated life expectancy with and without disability at age 65 according to two levels of healthy lifestyle among 15 121 participants. Estimates of multivariate-adjusted hazard ratios for morbidity and mortality associated with healthy lifestyle adjusted for age, gender, region, BMI, marital status, hypertension, diabetes, heart disease, stroke/cerebrovascular disease, dyslipidaemia, and SES. **Panel A.** Five disabilities in combination. **Panel B.** BADL disability. **Panel C.** Mobility disability. **Panel D.** Visual disability. **Panel** **E.** Hearing disability. **Panel F.** Cognitive disability.

Among older adults, a favourable lifestyle was associated with longer total LE, longer DFLE, and a greater percentage of LE free of five disabilities (**Figure 1** and Table S8 in the **Online Supplementary Document**). At age 65, individuals with unfavourable lifestyle levels had 12.828 (95% CI = 12.831–13.453) years of the estimated total LE and 7.279 (95% CI = 7.277–7.777) years of DFLE, whereas others had 17.187 (95% CI = 17.183–17.775) years of total LE and 10.243 (95% CI = 10.248–10.702) years of DFLE (**Table 2**). In addition, the percentage of LE without five disabilities in the unfavourable and favourable groups was 56.74% (95% CI = 55.65–57.83) and 59.60 (95% CI = 58.46–60.73) (**Table 2**).

**Table 2:** Baseline characteristics according to two levels of healthy lifestyle, presented as years (95% CI)

	Total LE at 65	Total difference	Free of five disabilities LE at 65	Free of five disabilities difference	Percentage of total
All participants (n = 15 121)*					
*Unfavourable group*	12.828 (12.831–13.453)	ref.	7.279 (7.277–7.777)	ref.	56.74 (55.65–57.83)
*Favourable group*	17.187 (17.183–17.775)	4.359 (3.447–5.271)	10.243 (10.248–10.702)	2.964 (2.287–3.641)	59.60 (58.46–60.73)
Males (n = 6492)†					
*Unfavourable group*	11.866 (11.866–12.724)	ref.	7.710 (7.715,8.405)	ref.	64.98 0(63.36–66.61)
*Favourable group*	14.496 (14.495–15.545)	2.630 (1.169,4.091)	9.914 (9.923,10.722)	2.204 (1.111,3.297)	68.38 (66.77–70.00)
Females (n = 8629)†					
*Unfavourable group*	13.818 (13.871–14.791)	ref.	6.622 (6.628–7.388)	ref.	47.92 (46.47–49.36)
*Favourable group*	18.689 (18.695–19.424)	4.871 (3.463–6.279)	10.301 (10.315–10.864)	3.679 (2.721–4.637)	55.12 (53.58–56.66)

CI – confidence interval, LE – life expectancy, ref – reference

*All LEs have been calculated with hazard ratios adjusted for age, gender, region, BMI, marital status, hypertension, diabetes, heart disease, stroke/cerebrovascular disease, dyslipidaemia, and SES.

†All LEs have been calculated with hazard ratios adjusted for age, region, BMI, marital status, hypertension, diabetes, heart disease, stroke/cerebrovascular disease, dyslipidaemia, and SES.

**Figure 2:**
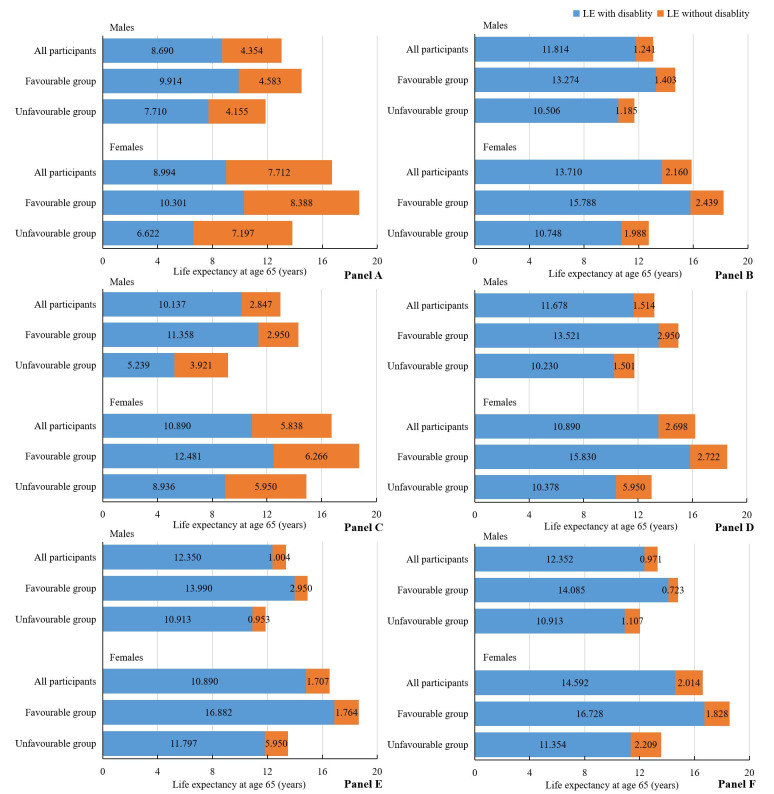
Estimated life expectancy with and without disability at age 65 according to two levels of healthy lifestyle for 6492 males and 8629 females, respectively. Estimates of multivariate-adjusted hazard ratios for morbidity and mortality associated with healthy lifestyle adjusted for age, region, BMI, marital status, hypertension, diabetes, heart disease, stroke/cerebrovascular disease, dyslipidaemia, and SES. **Panel A.** Five disabilities in combination. **Panel B.** BADL disability. **Panel C.** Mobility disability. **Panel D.** Visual disability. **Panel E.** Hearing disability. **Panel F.** Cognitive disability.

Equivalently, males with favourable lifestyle levels lived 2.630 (95% CI = 1.169–4.091) and 2.204 (95% CI = 1.111–3.297) more years of total LE and DFLE at age 65, compared with the unfavourable lifestyle group. The corresponding longer total LE and DFLE years for females at age 65 were 4.871 (95% CI = 3.463–6.279) and 3.679 (95% CI = 2.721–4.637) years (**Figure 2**, **Table 2**). Notably, although males had shorter total LE than females for both lifestyle levels, their DFLE was a higher proportion of total LE, which were 64.98% (95% CI = 63.36–66.61) and 68.38% (95% CI = 66.77–70.00%) among males with unfavourable and favourable lifestyle levels, and were 47.92% (95% CI = 46.47–49.36%) and 55.12% (95% CI = 53.58–56.66%) for females.

In a joint analysis of the association of SES and lifestyle with estimated LEs, compared to older adults with low SES and unfavourable lifestyle level (DFLE/total LE = 55.08%; 95% CI = 54.29–55.87), those with lower SES and favourable lifestyle level had more than 4.728 (95% CI = 3.777–5.679) years of total LE and 3.770 (95% CI = 3.018–4.522) years of DFLE at age 65 (DFLE/total LE = 61.81%; 95% CI = 61.04–62.59), those with higher SES and unfavourable lifestyle level had more than 2.399 (95% CI = 1.363–3.435) and 1.944 (95% CI = 1.151–2.737) years (DFLE/total LE = 59.23%; 95% CI = 58.45–60.02), as well as those with higher SES and favourable lifestyle level had more than 6.421 (95% CI = 5.542–7.300) and 5.102 (95% CI = 4.424–5.780) years (DFLE/total LE = 63.31%; 95% CI = 62.55–64.08).

### Life expectancy and years lived with and without the individual disability

At age 65, LE free of BADL disability, mobility disability, visual disability, hearing disability, and cognitive disability was 11.959 (95% CI = 11.371–12.504), 12.680 (95% CI = 11.990–13.302), 12.307 (95% CI = 11.763–12.816), 12.547 (95% CI = 11.940–13.125), and 12.714 (95% CI = 12.101–13.281) years among older adults adhering to unfavourable lifestyle, respectively (**Figure 1**, Table S9 in the **Online Supplementary Document**). Compared to the unfavourable group, participants gained 4.389 (95% CI = 3.679–5.146) more years without BADL disability, 3.421 (95% CI = 2.705–4.137) more years without mobility disability, 4.661 (95% CI = 3.954–5.368) more years without visual disability, 4.584 (95% CI = 3.837–5.342) more years without hearing disability, and 4.616 (95% CI = 3.858–5.374) more years without cognitive disability (**Figure 1**, Table S9 in the **Online Supplementary Document**). Additionally, unfavourable lifestyle levels were associated with a shorter percentage of individual DFLE. In the unfavourable and favourable groups, the percentage of LE without mobility disability was 68.35% (95% CI = 67.33–69.38) and 70.87% (95% CI = 69.82–71.92), 83.61% (95% CI = 82.8–84.43) and 87.05% (95% CI = 86.28–87.83%) for those without visual disability, 89.38% (95% CI = 88.70–90.05) and 91.65% (95% CI = 91.01–92.29) for those without hearing disability, as well as 87.42% (95% CI = 86.69–88.15) and 91.85% (95% CI = 91.22–92.48) for those without cognitive disability, respectively. However, there was no significant difference in the proportion of LE without BADL disability between unfavourable lifestyle (87.57%; 95% CI = 86.84–88.29) and favourable lifestyle groups (87.94%; 95% CI = 87.19–88.70).

**Figure 3:**
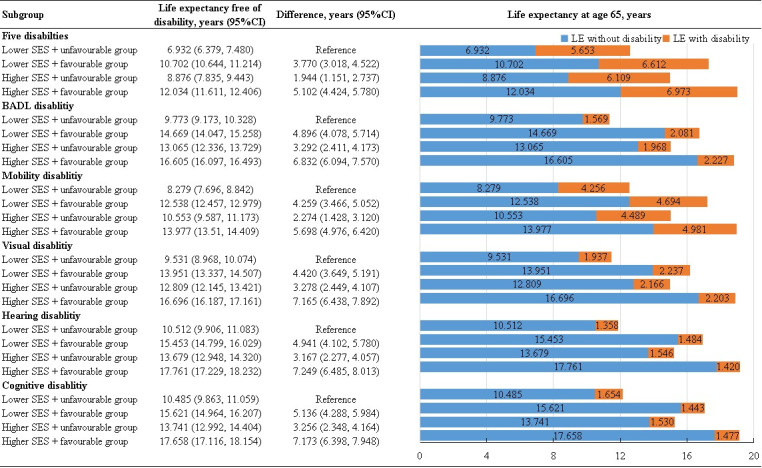
Association of healthy lifestyle and socioeconomic status with estimated life expectancy with and without disability at age 65. Estimates of multivariate adjusted hazard ratios for morbidity and mortality associated with healthy lifestyle adjusted for age, gender, region, BMI, marital status, hypertension, diabetes, heart disease, stroke/cerebrovascular disease, and dyslipidaemia.

Corresponding associations were also found separately in each of the five individual disability by gender and SES analysis. Among males at age 65, favourable lifestyles were associated with 2.768 (95% CI = 1.636–3.900) years’ longer LE without BADL disability, 2.398 (95% CI = 1.233–3.563) more years without mobility disability, 3.291 (95% CI = 2.221–4.456) more years without visual disability, 3.077 (95% CI = 1.941–4.242) more years without hearing disability, and 3.172 (95% CI = 2.038–4.306) more years without cognitive disability, respectively. Compared with unfavourable group, females adhering to favourable lifestyles gained 5.040 (95% CI = 3.976–6.104) years’ longer LE without BADL disability, 3.545 (95% CI = 2.702–4.388) more years without mobility disability, 5.452 (95% CI = 4.474–6.295) more years without visual disability, 5.085 (95% CI = 4.0416–5.928) more years without hearing disability, and 5.374 (95% CI = 4.314–6.434) more years without cognitive disability, respectively (**Figure 2**, Table S9 in the **Online Supplementary Document**). When we analysed the joint association of SES and lifestyle, compared to the group with lower SES and unfavourable lifestyle level, the other three groups lived between 3.292 and 6.832 more years without BADL disability, 2.274 to 5.698 more years without mobility disability, 3.278 to 7.165 more years without visual disability, 3.167 to 7.249 more years without hearing disability, and 3.256 to 7.173 more years without cognitive disability at age 65 (**Figures 2** and **Figure** **3**, Table S9 in the **Online Supplementary Document**).

### Sensitivity analysis

In sensitivity analyses 1 and 2 by different inclusion criteria, we observed consistent associations between healthy lifestyles and prolonged total LE and the proportion of DFLE at age 65 years (Tables S10 and S11 in the **Online Supplementary Document**). We also estimated the LEs related to each lifestyle factor separately. Never smoking, never drinking, regular physical exercise, a healthy diet, active cognitive activity, and healthy sleep were all associated with a greater percentage of LE free of five disabilities in combination. However, the association of each healthy lifestyle with longer total LE and DFLE were also not identical.

## DISCUSSION

To our knowledge, the current study provides the first comprehensive analysis of a combination of multiple lifestyle factors and LE free of five major disabilities. We found that adherence to healthy lifestyles was associated with longer total LE, longer DFLE, and a greater percentage of LE free of five disabilities. The total LE at age 65 in unfavourable and favourable lifestyle groups were 12.828 and 17.187 years, of which 56.74% (DFLE = 7.279 years) and 59.60% (DFLE = 10.243 years) were spent without five major disabilities in combination. In addition, males and females with favourable lifestyle levels had both longer total LE (14.496–18.689 years) and greater proportion of their DFLE (68.38% and 55.12%). Furthermore, the joint analysis of SES and lifestyle showed that all three other groups had longer total LE, longer DFLE and a higher proportion of DFLE at age 65 compared to the low SES and unfavourable lifestyle group.

Due to regional and cultural traditions, lifestyles vary by country and population. Still, our findings are consistent with previous findings from different countries on the association of the individual or clustering effect of lifestyle-related risk factors on LE with and without disability. Mehta et al. found that participants aged 50 years who had never smoked had DFLE (self-reported need for help or inability to perform BADL) of 5.6 years (males) and 5.3 years (females) higher than their peers who had ever smoked [16]. A large study in Australia showed that low-educated females with three unhealthy lifestyle factors (obesity, smoking, and no exercise) had 6.4 years of fewer DFLE (short form-36 scores below 40 in the physical functioning domain and requiring regular help with daily tasks due to long-term illness, disability or frailty) at age 70 than high educated women with healthy lifestyle factors in the 1921–26 cohort [14]. The Ohsaki cohort study indicated an inverse U-shaped association between sleep duration and DFLE (self-reported need for help or inability to perform BADL) at age 65 [15]. Similarly, for other chronic diseases, males and females who adopted four or five healthy lifestyle factors (a diet for brain health, late-life cognitive activities, moderate or vigorous physical activity, no smoking, and light to moderate alcohol consumption) had approximately 6.4 and 4.5 years longer LEs free of Alzheimer dementia at age 65 compared with those who adopted zero or one healthy lifestyle factors in the Chicago Health and Aging Project [10]. The Nurses’ Health Study and the Health Professionals Follow-Up Study found that four or five healthy lifestyle factors were associated with longer LE at age 50 free of three major chronic diseases in combination (cancer, cardiovascular disease, and type 2 diabetes) of approximately 7.6 and 10.7 years for males and females, compared to those with no healthy lifestyle factor [12]. Taken together, these results indicate that a favourable lifestyle extended more DFLE, thereby occupying the space for periods with disabilities, leading to a compression of years of life lived with disabilities.

Our study extends previous findings by comprehensively assessing six lifestyle factors and five major disabilities individually and in combination. We observed a relatively shorter gain in LE free of mobility disability associated with an unfavourable lifestyle than the gained LE free of BADL, visual, hearing or cognitive disability, which may be partially caused by the different preventable attribution fractions for lifestyle related to a specific disability. For each lifestyle, six factors were significantly associated with a greater percentage of LE free of five disabilities in combination, but the other correlations about LEs were not identical. The overall effect of multiple modifiable lifestyle factors may synergistically affect mortality risk [28]. Compared to other high-income countries [10,13], older Chinese adults may have less dietary diversity and engage in less regular exercise [29,30]. Still, studies in different-income countries have shown that healthy lifestyles can prolong HALE. As total LE cannot be extended indefinitely, these findings had important implications for the improvement of healthy ageing. Public policies for improving food and the physical environment conducive to adopting a healthy diet and lifestyle, as well as relevant policies and regulations (for example, smoking ban in public places, trans-fat restrictions, or provision of places to exercise and recreation), are essential to improving total LE, especially LE free of major disabilities.

Both gender and SES subgroup results also suggested that adherence to a favourable lifestyle not only prolongs life span but also improves the quality of ageing (disability-free). Intriguingly, we observed that females lived an average of 3.662 years longer in total but experienced only 0.304 more years of DFLE than males, resulting from the higher risk of disability onset and lower risk of death in females compared to males, as observed in our study, which possibly because more females have a low level of education as well as being economically dependent. Gender differences in healthy LE have also been found in previous studies [7,18], more research on the development of DFLE by gender is needed to understand further what is driving the gender differences in DFLE observed in older adults. Moreover, given the vital role of SES in early morbidity and mortality, we performed joint analyses of combined socioeconomic indicators and lifestyle with estimated LEs. Consistent with previous studies [14,17–19], we found that a lower SES was associated with a shorter DFLE. Of interest, the joint analyses showed that a favourable lifestyle might offset disparities in DFLE due to lower SES. This finding indicated that the gap in LEs between lower SES and others might be narrowed by improving healthy lifestyles and could contribute to reducing health inequalities. Because estimates of LEs consider both morbidity and mortality, the results of subgroup analyses can be helpful indicators for health professionals and the general public and allow policymakers to better estimate future health care costs and plan for health care needs.

The major strength of this study is that it analysed longitudinal data and used multi-state models to take into account dynamic changes in lifestyle factors over time and the time of transition between any two states (including disability that can be converted to health with access to better medical coverage (hearing aids or rehabilitation training)). The large sample size further allowed us to explore effect sizes with sufficient statistical power. This study also has several limitations. First, the assessments of five major disabilities mainly focused on whether the functionality could affect the daily life of the elderly. Thus, the measurements could not directly compare with clinical standards. This convenient form could be easily implemented and quickly screened among older adults to evaluate functional impairments in a large-scale, population-based cohort study. Second, self-reported questionnaire assessing lifestyle factors could be subject to measurement error, and these criteria are susceptible to bias in participant reporting due to the lack of food intake portions and duration of cognitive activities. Although some studies utilised self-reported lifestyle data, it may still affect the conclusion's validity. Third, some participants were excluded due to missing data or not returned for follow-up evaluations, which may have led to selection bias. Fourthly, residual and unmeasured confounding might also exist even though we controlled for a wide range of potential confounders (e.g. region, marital status, and SES). Fifthly, our study only included older Chinese adults from the CLHLS, so the findings may not be generalisable to other populations, but this does provide an opportunity to make both national and international comparisons. Lastly, the life tables created in this study were logged only from the age of 65, and the onset of the disabilities could occur earlier in life. Therefore, further longitudinal studies should be conducted to investigate broadly the impact of objective criteria for healthy lifestyles on LE free from disabilities by clinical standards across the lifespan and to track the direct causal impact of lifestyle changes over time on the development of disabilities.

## CONCLUSIONS

This study indicated that a favourable lifestyle was associated with longer total LE, longer LE free of five major disabilities and a higher proportion of DFLE, which may also contribute to narrowing socioeconomic health inequalities. To address healthy ageing and reduce the care burden, policymakers should adopt relevant policies and regulations to improve food and the physical environment conducive to adherence to a healthy diet and lifestyle, as well as expand rehabilitation services in primary health care to prolong DFLE in older adults, especially in LMICs.

## Additional material


Online Supplementary Document
