# Supplementary material for: Association of healthy lifestyle with life expectancy free of five major disabilities in Chinese older adults
Source: J Glob Health. 2024 Jan 12;14:04034. doi: 10.7189/jogh.14.04034 (PMC10785201; doi:10.7189/jogh.14.04034)
Supplement: Online Supplementary Document [file jogh-14-04034-s001.pdf]

Online Supplementary Document

Details for statistical analysis 1
Table S1. The Chinese version of the mini-mental state examination adopted in the Chinese Longitudinal Healthy Longevity Study 3
Table S2. The socioeconomic vulnerability index scoring details adopted in this study 4
Table S3. Bayesian information criterion values for different link functions and degrees of different variable 5
Table S4. Posterior probabilities of latent classes with different variables 6
Table S5. Distribution of missing values with different follow-up years 7
Table S6. Baseline characteristics according to two levels of healthy lifestyle in males and females 8
Table S7. Baseline characteristics according to two levels of healthy lifestyle in lower and higher SES group 10
Table S8. Hazard ratios and 95% confidence intervals for transitions between five major disabilities states and death 12
Table S9. Total life expectancy and years lived without five disabilities by lifestyle level 17
Table S10. Total life expectancy and years lived with and without disability at age 65 (95%CI) by two levels of healthy lifestyle among 13138 participants at baseline in 2008 20
Table S11. Total life expectancy and years lived with and without disability by two levels of healthy lifestyle among 11853 participants without any of the five disabilities at baseline 23
Figure S1. Flow Chart across 4 waves of the CLHLS from 2008 to 2018 26
Figure S2. Trajectories of healthy lifestyle and SEVI predicted by the LCGMM among 15121 older adults. 27
Figure S3. Markov multistate transition model over the time of follow-up applied to the multistate life table for the estimation of life expectancy 28
Figure S4. Estimated life expectancy (LE) with and without disability at age 65 according to levels of individual lifestyle factors among 15121 participants.. 29

Details for statistical analysis

Assuming a ratio of 40%: 60% for the size of people in the favourable versus unfavourable lifestyle group, with a p-value of less than 0.05, a power of 90%, an OR of 0.7, and the disability/death rate of 30% in the favourable group [3,21], we calculated the sample size to be at least 720 for the favourable group and 1080 for the unfavourable group.

LCGMM was applied to model healthy lifestyle and SES longitudinal trajectories over the follow-up period in the older adults and to identify distinct subgroups following similar patterns. We compared two- to five-class LCGMM models iterating 1st- to 3rd-degree fractional polynomials. The best-fitting one was determined by the minimum absolute value of the bayesian information criterion (BIC), the average posterior probability (AvePP) of each subgroup not less than 0.7, the proportion of each subgroup with posterior probability greater than 0.7 not less than 0.65, and the membership of each subgroup not less than 5% of the total population (Table S3 and Table S4 in the Online Supplementary Document). Missing data for covariates were filled through multiple imputation methods (Table S5 in the Online Supplementary Document). Data were assumed to be missing at random, with predictive mean matching method used for missing continuous variables and Logistic regression model for missing binary variables. If distributed normally by the Kolmogorov-Smirnov test, continuous variables were presented as means and standard deviations; otherwise, medians and interquartile ranges were applied. Categorical variables were presented by numbers and proportions. Baseline characteristics were compared between subjects in different estimated healthy lifestyle trajectory classes using independent samples t test for continuous variables if distributed normally or Mann-Whitney U test if not, and using chi-square tests or Fisher's exact tests for categorical variables.

Population-based multistate life tables were used to calculate the total LE and years lived with and without the five major disabilities. We built a total of six multistate life tables, one with a combination of the disabilities in BADL, mobility, vision, hearing and cognition and five with individual disabilities. To assess the association between healthy lifestyle and LE free of five major disabilities, we took into account three states (free of disability, presence of disability, and death), and four transitions between states (from non-disability to incident disability, from non-disability to mortality, from disability to mortality, as well as from disability to non-disability) in a Markov multistate transition model (Figure S 3). First, we calculated the overall transition rates for each transition using survival analysis with Gompertz distribution. Second, hazard ratios (HRs) by healthy lifestyle were calculated for each transition in Markov multistate transition models. The models were adjusted by age, gender, region, BMI, marital status, medical illnesses (hypertension, diabetes, heart disease, stroke and other cerebrovascular, and dyslipidemia), and SES. Third, we calculated proportion of lifestyle levels among the sub-study population for each transition. Finally, we combined overall transition rates, HRs and the proportion of lifestyle levels to derive weighted transition rates, which were used to create multistate life tables to calculate total LE and LE with and without disabilities for each group. The multistate life table started at age 65 years and ended at age 115 years. The 95% confidence intervals (CIs) of LE estimation were calculated using bootstrapping with 1000 iterations. Considering the pathophysiological differences between genders on lifestyle, the analysis was repeatedly stratified by gender (male and female). In addition, we conducted a joint analysis of the relationship between SES and lifestyles on the DFLE, as lower SES has a opposite impact on the DFLE.

Three additional sensitivity analyses were conducted to evaluate the robustness of our findings. First, we re-performed the original analysis by including only participants whose baseline wave was 2008 (n=13138). Second, we excluded participants who already had any of the five disabilities at baseline

from the 15121 participates (n=11853) and repeated the main analysis. Finally, the association of each single lifestyle factor with LE free of five major disabilities were also evaluated in models.

All statistical analysis was conducted by using R software (version 4.2.3, R Core Team, R Foundation for Statistical Computing, Vienna, Austria). The following packages were used: mice for imputing, lcmm for LCGMM, msm for Markov multistate transition models, and elect for LE estimation using multistate models. Statistical significance was defined by $p<0.05$ in two-sided testing.

Table S1. The Chinese version of the mini-mental state examination adopted in the Chinese Longitudinal Healthy Longevity Study

Domain	Question	Score (Total = 30)
Orientation	What time of day is it right now (morning, afternoon, evening)?	1
	What is the animal year of this year?	1
	What is the date (day and month) of the mid-autumn festival?	1
	What is the season right now?	1
	What is the name of this county or district?	1
	Please name as many kinds of food as possible in 1 minute (1 point for each food and 7 points for those who name 7 or more foods).	7
Registration	Table (1 point), apple (1 point), cloth (1 point). Please repeat these 3 objects.	3
Attention and calculation	I will ask you to spend \$3 from \$20, then you must spend \$3 from the number you arrived at and continue to spend \$3 until you are asked to stop (1 point for each calculation).	5
Recall	The individual is asked to draw a figure of overlapping pentagons.	1
	Name the 3 objects learned earlier (table, apple, and cloth).	3
Language	Naming pen (1 point) and watch (1 point).	2
	Repeating the following sentence: "What you plant is what you will get."	1
	The individual is asked to follow the interviewer's instruction: "Take the paper using your right hand (1 point), fold it in the middle using both hands (1 point), and place the paper on the floor (1 point)."	3

Note: We scored each question as zero (wrong or unable to answer) or one (correct) , and the scores ranged between 0 and 30, with a higher score implying better cognitive performance.

Table S2. The socioeconomic vulnerability index scoring details adopted in this study

Question	Answers	Score (Total = 6)
Years of education	7+ years of schooling	0
	1–6 years of schooling	0.5
	0 years of schooling	1
Primary occupation	White collar (professional technician / doctor / teacher, office worker, and military)	0
	Other types	1
Daily expenses paid primarily by own salary or pension	Yes	0
	No	1
Family economic status	Very rich	0
	Rich	0.25
	General	0.5
	Poor	0.75
	Very poor	1
Timely access to healthcare services	Yes	0
	No	1
Place of residence	Urban	0
	Town	0.5
	Rural	1

Note: Socioeconomic vulnerability index score was obtained by dividing the total score of the above variables by six, ranging from 0 to 1, with higher score indicating lower levels of socioeconomic status.

Table S3. Bayesian information criterion values for different link functions and degrees of different variable

Link function	Degree	Number of latent classes			
		2	3	4	5
Healthy lifestyle					
Linear	1	19932.200	19960.810	19987.282	20007.279
	2	19951.462	19989.765	20027.570	20060.137
	3	19965.650	20013.868	20059.977	20100.511
Beta	1	19155.522	19188.895	19228.314	19267.065
	2	19264.815	19312.919	19404.536	19496.224
	3	19334.687	19418.438	19484.410	19609.440
Splines	1	**18848.018**	**18884.172**	**18924.476**	18993.425
	2	**18912.445**	**18968.572**	19085.984	19199.986
	3	19222.164	19489.759	19750.746	19955.888
SEVI					
Linear	1	-69236.527	-69255.103	-69297.831	-69328.237
	2	-63907.284	-63748.887	-65816.026	-68245.189
	3	-63387.997	-68202.831	-67179.107	-65054.943
Beta	1	-65379.538	-65354.764	-65320.517	-65275.166
	2	-65364.877	-65337.042	-65286.745	-65238.323
	3	-65171.224	-65122.010	-65079.129	-65008.707
Splines	1	**-70413.333**	**-71245.818**	**-69799.758**	-67582.987
	2	-67568.043	**-69804.263**	**-69765.845**	-69703.508
	3	-67255.489	-66545.406	-66623.852	-66093.106

SEVI: socioeconomic vulnerability index

Table S4. Posterior probabilities of latent classes with different variables

Model	BIC	Average posterior probabilities in each class	Posterior probabilities > 0.7 (%)	Minimum subgroup (%)
Healthy lifestyle				
**Model 1**	**18848.018**	**0.717, 0.702**	**78.64, 70.88**	**42.00**
Model 2	18884.172	0.723, 0.686, 0.738	50.35, 37.61, 93.33	0.30
Model 3	18912.445	0.764, 0.719	68.44, 54.06	33.81
Model 4	18924.476	0.752, 0.614, 0.769, 0.695	66.72, 47.65, 74.38, 67.32	0.54
Model 5	18968.572	0.722, 0.735, 0.721	64.95, 50.13, 75.51	0.32
SEVI				
Model 1	-71245.818	0.823, 0.966, 0.917	75.76, 97.19, 91.91	3.43
**Model 2**	**-70413.333**	**0.716, 0.739**	**80.52, 86.59**	**40.94**
Model 3	-69804.263	Na, 0.979, 0.869	Na, 98.00, 79.20	0.00
Model 4	-69799.758	0.813, 0.834, 0.757, 0.892	75.34, 84.22, 60.17, 90.16	3.36
Model 5	-69765.845	0.818, 0.784, 0.784, 0.869	88.39, 68.48, 65.69, 80.46	5.12

Note: *P* values for all parameters in the models fitted by the maximum likelihood method were less than 0.05.

BIC: bayesian information criterion.

Table S5. Distribution of missing values with different follow-up years

Characteristics	2008(n=13138)	2011(n=8622)	2014(n=6063)	2018(n=3004)
Region	0.33%	0.53%	0.97%	1.17%
Marital status	0.00%	0.28%	1.06%	0.93%
BMI (kg/m^2)	0.00%	3.79%	2.08%	0.23%
Hypertension	0.02%	0.36%	0.92%	0.13%
Diabetes	0.01%	0.27%	0.87%	0.13%
Heart disease	0.00%	0.24%	0.82%	0.17%
Stroke, cerebrovascular disease	0.01%	0.20%	0.79%	0.13%
Dyslipidemia	0.02%	0.66%	1.42%	0.23%
Occupation	0.04%	0.48%	1.37%	1.66%
Economic independence	0.00%	0.26%	0.66%	2.93%
Economic status	0.00%	0.96%	1.11%	1.03%
Timely access to healthcare services	0.00%	0.53%	0.64%	1.26%

BMI: body mass index.

Table S6. Baseline characteristics according to two levels of healthy lifestyle in males and females

Characteristics	Males			Females		
	Total (n=6492)	Favourable group (n=3181)	Unfavourable group (n=3311)	Total (n=8629)	Favourable group (n=4016)	Unfavourable group (n=4613)
Age (years)	84.53±10.50	82.62±10.38	86.38±10.29	89.96±11.42	86.02±11.94	93.39±9.73
Region						
East	3138 (48.34%)	1688 (53.07%)	1450 (43.79%)	4025 (46.65%)	2137 (53.21%)	1888 (40.93%)
Centre	1697 (26.14%)	787 (24.74%)	910 (27.48%)	2390 (27.70%)	999 (24.88%)	1391 (30.15%)
West	1657 (25.52%)	706 (22.19%)	951 (28.72%)	2214 (25.66%)	880 (21.91%)	1334 (28.92%)
BMI (kg/m^2)						
＜18.5	1679 (25.86%)	661 (20.78%)	1018 (30.75%)	3354 (38.87%)	1238 (30.83%)	2116 (45.87%)
18.5 and ＜24	3784 (58.29%)	1849 (58.13%)	1935 (58.44%)	4142 (48.00%)	2060 (51.29%)	2082 (45.13%)
≥24 and ＜28	844 (13.00%)	542 (17.04%)	302 (9.12%)	879 (10.19%)	569 (14.17%)	310 (6.72%)
≥28	185 (2.85%)	129 (4.06%)	56 (1.69%)	254 (2.94%)	149 (3.71%)	105 (2.28%)
Marital status						
Married	3200 (49.29%)	1819 (57.18%)	1381 (41.71%)	1533 (17.77%)	1064 (26.49%)	469 (10.17%)
Widowed, divorced, separated, or single	3292 (50.71%)	1362 (42.82%)	1930 (58.29%)	7096 (82.23%)	2952 (73.51%)	4144 (89.83%)
Medical illnesses						
Hypertension	1267 (19.52%)	686 (21.57%)	581 (17.55%)	1851 (21.45%)	998 (24.85%)	853 (18.49%)
Diabetes	176 (2.71%)	116 (3.65%)	60 (1.81%)	177 (2.05%)	125 (3.11%)	52 (1.13%)
Heart disease	533 (8.21%)	324 (10.19%)	209 (6.31%)	778 (9.02%)	456 (11.35%)	322 (6.98%)
Stroke, cerebrovascular disease	479 (7.38%)	238 (7.48%)	241 (7.28%)	440 (5.10%)	219 (5.45%)	221 (4.79%)
Dyslipidemia	105 (1.62%)	74 (2.33%)	31 (0.94%)	104 (1.21%)	60 (1.49%)	44 (0.95%)
Healthy lifestyle factors						
Healthy diet	3445 (53.07%)	2242 (70.48%)	1203 (36.33%)	4074 (47.21%)	2721 (67.75%)	1353 (29.33%)
Never smoking	4310 (66.39%)	2499 (78.56%)	1811 (54.70%)	8157 (94.53%)	3898 (97.06%)	4259 (92.33%)
Regular physical exercise	2151 (33.13%)	1572 (49.42%)	579 (17.49%)	1730 (20.05%)	1348 (33.57%)	382 (8.28%)
Never drinking	4581 (70.56%)	2569 (80.76%)	2012 (60.77%)	7903 (91.59%)	3815 (95.00%)	4088 (88.62%)
Active cognitive activity	4560 (70.24%)	2832 (89.03%)	1728 (52.19%)	4288 (49.69%)	3093 (77.02%)	1195 (25.91%)
Healthy sleep	3062 (47.17%)	1958 (61.55%)	1104 (33.34%)	3591 (41.62%)	2360 (58.76%)	1231 (26.69%)
The number of healthy lifestyle factors	3.41±1.30	4.30±0.96	2.55±0.96	3.45±1.14	4.29±0.87	2.71±0.78
SES group						
Lower	2765 (42.59%)	954 (29.99%)	1811 (54.70%)	6166 (71.46%)	2380 (59.26%)	3786 (82.07%)
Higher	3727 (57.41%)	2227 (70.01%)	1500 (45.30%)	2463 (28.54%)	1636 (40.74%)	827 (17.93%)

SES factors						
Years of education						
<1	2436 (37.52%)	942 (29.61%)	1494 (45.12%)	7227 (83.75%)	3054 (76.05%)	4173 (90.46%)
1~6	2976 (45.84%)	1513 (47.56%)	1463 (44.19%)	1128 (13.07%)	735 (18.30%)	393 (8.52%)
＞6	1080 (16.64%)	726 (22.82%)	354 (10.69%)	274 (3.18%)	227 (5.65%)	47 (1.02%)
Occupation						
White collar	843 (12.99%)	612 (19.24%)	231 (6.98%)	191 (2.21%)	162 (4.03%)	29 (0.63%)
Other types	5649 (87.01%)	2569 (80.76%)	3080 (93.02%)	8438 (97.79%)	3854 (95.97%)	4584 (99.37%)
Economic independence						
One's own	2466 (37.99%)	1542 (48.48%)	924 (27.91%)	1068 (12.38%)	793 (19.75%)	275 (5.96%)
Others	4026 (62.01%)	1639 (51.52%)	2387 (72.09%)	7561 (87.62%)	3223 (80.25%)	4338 (94.04%)
Economic status						
Very rich	82 (1.26%)	55 (1.73%)	27 (0.82%)	79 (0.92%)	54 (1.34%)	25 (0.54%)
Rich	895 (13.79%)	571 (17.95%)	324 (9.79%)	988 (11.45%)	585 (14.57%)	403 (8.74%)
General	4450 (68.55%)	2241 (70.45%)	2209 (66.72%)	5960 (69.07%)	2879 (71.69%)	3081 (66.79%)
Poor	870 (13.40%)	271 (8.52%)	599 (18.09%)	1301 (15.08%)	422 (10.51%)	879 (19.05%)
Very poor	195 (3.00%)	43 (1.35%)	152 (4.59%)	301 (3.49%)	76 (1.89%)	225 (4.88%)
Timely access to healthcare services						
Yes	6084 (93.72%)	3083 (96.92%)	3001 (90.64%)	7940 (92.02%)	3841 (95.64%)	4099 (88.86%)
No	408 (6.28%)	98 (3.08%)	310 (9.36%)	689 (7.98%)	175 (4.36%)	514 (11.14%)
Place of residence						
Urban	994 (15.31%)	710 (22.32%)	284 (8.58%)	1221 (14.15%)	829 (20.64%)	392 (8.50%)
Town	1294 (19.93%)	710 (22.32%)	584 (17.64%)	1679 (19.46%)	837 (20.84%)	842 (18.25%)
Rural	4204 (64.76%)	1761 (55.36%)	2443 (73.78%)	5729 (66.39%)	2350 (58.52%)	3379 (73.25%)
SEVI	0.57±0.20	0.50±0.21	0.63±0.16	0.69±0.14	0.64±0.16	0.73±0.11
Disability						
BADL	942 (14.51%)	315 (9.90%)	627 (18.94%)	2326 (26.96%)	705 (17.55%)	1621 (35.14%)
Mobility	2343 (36.09%)	849 (26.69%)	1494 (45.12%)	5234 (60.66%)	1839 (45.79%)	3395 (73.60%)
Vision	940 (14.48%)	307 (9.65%)	633 (19.12%)	2339 (27.11%)	684 (17.03%)	1655 (35.88%)
Hearing	1223 (18.84%)	387 (12.17%)	836 (25.25%)	2823 (32.72%)	807 (20.09%)	2016 (43.70%)
Cognition	1221 (18.81%)	337 (10.59%)	884 (26.70%)	3241 (37.56%)	910 (22.66%)	2331 (50.53%)

BMI: body mass index; SES: socioeconomic status; SEVI: socioeconomic vulnerability index; BADL: basic activity of daily living.

Table S7. Baseline characteristics according to two levels of healthy lifestyle in lower and higher SES group

Characteristics	Lower SES			Higher SES		
	Total (n=8931)	Favourable group (n=3334)	Unfavourable group (n=5597)	Total (n=6190)	Favourable group (n=3863)	Unfavourable group (n=2327)
Age (years)	90.58±10.24	87.61±10.80	92.35±9.46	83.38±11.54	81.85±11.23	85.92±11.60
Gender						
Male	2765 (30.96%)	954 (28.61%)	1811 (32.36%)	3727 (60.21%)	2227 (57.65%)	1500 (64.46%)
Female	6166 (69.04%)	2380 (71.39%)	3786 (67.64%)	2463 (39.79%)	1636 (42.35%)	827 (35.54%)
Region						
East	3957 (44.31%)	1713 (51.38%)	2244 (40.09%)	3206 (51.79%)	2112 (54.67%)	1094 (47.01%)
Centre	2582 (28.91%)	884 (26.51%)	1698 (30.34%)	1505 (24.31%)	902 (23.35%)	603 (25.91%)
West	2392 (26.78%)	737 (22.11%)	1655 (29.57%)	1479 (23.89%)	849 (21.98%)	630 (27.07%)
BMI (kg/m^2)						
＜18.5	3539 (39.63%)	1106 (33.17%)	2433 (43.47%)	1494 (24.14%)	793 (20.53%)	701 (30.12%)
18.5 and ＜24	4420 (49.49%)	1742 (52.25%)	2678 (47.85%)	3506 (56.64%)	2167 (56.10%)	1339 (57.54%)
≥24 and ＜28	762 (8.53%)	379 (11.37%)	383 (6.84%)	961 (15.53%)	732 (18.95%)	229 (9.84%)
≥28	210 (2.35%)	107 (3.21%)	103 (1.84%)	229 (3.70%)	171 (4.43%)	58 (2.49%)
Marital status						
Married	1875 (20.99%)	924 (27.71%)	951 (16.99%)	2858 (46.17%)	1959 (50.71%)	899 (38.63%)
Widowed, divorced, separated, or single	7056 (79.01%)	2410 (72.29%)	4646 (83.01%)	3332 (53.83%)	1904 (49.29%)	1428 (61.37%)
Medical illnesses						
Hypertension	1705 (19.09%)	724 (21.72%)	981 (17.53%)	1413 (22.83%)	960 (24.85%)	453 (19.47%)
Diabetes	123 (1.38%)	56 (1.68%)	67 (1.20%)	230 (3.72%)	185 (4.79%)	45 (1.93%)
Heart disease	600 (6.72%)	269 (8.07%)	331 (5.91%)	711 (11.49%)	511 (13.23%)	200 (8.59%)
Stroke, cerebrovascular disease	455 (5.09%)	161 (4.83%)	294 (5.25%)	464 (7.50%)	296 (7.66%)	168 (7.22%)
Dyslipidemia	87 (0.97%)	42 (1.26%)	45 (0.80%)	122 (1.97%)	92 (2.38%)	30 (1.29%)
Healthy lifestyle factors						
Healthy diet	3615 (40.48%)	2087 (62.60%)	1528 (27.30%)	3904 (63.07%)	2876 (74.45%)	1028 (44.18%)
Never smoking	7721 (86.45%)	3102 (93.04%)	4619 (82.53%)	4746 (76.67%)	3295 (85.30%)	1451 (62.35%)
Regular physical exercise	1559 (17.46%)	1011 (30.32%)	548 (9.79%)	2322 (37.51%)	1909 (49.42%)	413 (17.75%)
Never drinking	7639 (85.53%)	3070 (92.08%)	4569 (81.63%)	4845 (78.27%)	3314 (85.79%)	1531 (65.79%)
Active cognitive activity	4078 (45.66%)	2465 (73.94%)	1613 (28.82%)	4770 (77.06%)	3460 (89.57%)	1310 (56.30%)
Healthy sleep	3646 (40.82%)	2059 (61.76%)	1587 (28.35%)	3007 (48.58%)	2259 (58.48%)	748 (32.14%)
The number of healthy lifestyle factors	3.16±1.13	4.14±0.84	2.58±0.84	3.81±1.23	4.43±0.94	2.79±0.91
SES factors						

Years of education						
<1	7508 (84.07%)	2744 (82.30%)	4764 (85.12%)	2155 (34.81%)	1252 (32.41%)	903 (38.81%)
1~6	1384 (15.50%)	572 (17.16%)	812 (14.51%)	2720 (43.94%)	1676 (43.39%)	1044 (44.86%)
＞6	39 (0.44%)	18 (0.54%)	21 (0.38%)	1315 (21.24%)	935 (24.20%)	380 (16.33%)
Occupation						
White collar	15 (0.17%)	4 (0.12%)	11 (0.20%)	1019 (16.46%)	770 (19.93%)	249 (10.70%)
Other types	8916 (99.83%)	3330 (99.88%)	5586 (99.80%)	5171 (83.54%)	3093 (80.07%)	2078 (89.30%)
Economic independence						
One's own	167 (1.87%)	55 (1.65%)	112 (2.00%)	3367 (54.39%)	2280 (59.02%)	1087 (46.71%)
Others	8764 (98.13%)	3279 (98.35%)	5485 (98.00%)	2823 (45.61%)	1583 (40.98%)	1240 (53.29%)
Economic status						
Very rich	37 (0.41%)	13 (0.39%)	24 (0.43%)	124 (2.00%)	96 (2.49%)	28 (1.20%)
Rich	607 (6.80%)	290 (8.70%)	317 (5.66%)	1276 (20.61%)	866 (22.42%)	410 (17.62%)
General	6213 (69.57%)	2494 (74.81%)	3719 (66.45%)	4197 (67.80%)	2626 (67.98%)	1571 (67.51%)
Poor	1629 (18.24%)	443 (13.29%)	1186 (21.19%)	542 (8.76%)	250 (6.47%)	292 (12.55%)
Very poor	445 (4.98%)	94 (2.82%)	351 (6.27%)	51 (0.82%)	25 (0.65%)	26 (1.12%)
Timely access to healthcare services						
Yes	7897 (88.42%)	3087 (92.59%)	4810 (85.94%)	6127 (98.98%)	3837 (99.33%)	2290 (98.41%)
No	1034 (11.58%)	247 (7.41%)	787 (14.06%)	63 (1.02%)	26 (0.67%)	37 (1.59%)
Place of residence						
Urban	79 (0.88%)	31 (0.93%)	48 (0.86%)	2136 (34.51%)	1508 (39.04%)	628 (26.99%)
Town	1324 (14.82%)	536 (16.08%)	788 (14.08%)	1649 (26.64%)	1011 (26.17%)	638 (27.42%)
Rural	7528 (84.29%)	2767 (82.99%)	4761 (85.06%)	2405 (38.85%)	1344 (34.79%)	1061 (45.60%)
SEVI	0.75±0.07	0.73±0.06	0.76±0.08	0.48±0.16	0.45±0.17	0.52±0.13
Disability						
BADL	2181 (24.42%)	514 (15.42%)	1667 (29.78%)	1087 (17.56%)	506 (13.10%)	581 (24.97%)
Mobility	5361 (60.03%)	1539 (46.16%)	3822 (68.29%)	2216 (35.80%)	1149 (29.74%)	1067 (45.85%)
Vision	2418 (27.07%)	601 (18.03%)	1817 (32.46%)	861 (13.91%)	390 (10.10%)	471 (20.24%)
Hearing	3028 (33.90%)	745 (22.35%)	2283 (40.79%)	1018 (16.45%)	449 (11.62%)	569 (24.45%)
Cognition	3374 (37.78%)	789 (23.67%)	2585 (46.19%)	1088 (17.58%)	458 (11.86%)	630 (27.07%)

BMI: body mass index; SES: socioeconomic status; SEVI: socioeconomic vulnerability index; BADL: basic activity of daily living.

Table S8. Hazard ratios and 95% confidence intervals for transitions between five major disabilities states and death

	State 0 - State 1	State 0 - State 2	State 1 - State 0	State 1 - State 2
**Five disabilities**				
Age (years)	1.060(1.055,1.065)	1.071(1.061,1.081)	0.946(0.938,0.954)	1.042(1.039,1.045)
Gender				
Male	Reference			
Female	1.193(1.090,1.305)	0.395(0.277,0.562)	0.796(0.693,0.914)	0.712(0.678,0.747)
Region				
East	Reference			
Centre	0.938(0.845,1.041)	2.304(1.588,3.341)	0.924(0.787,1.086)	0.989(0.938,1.042)
West	0.883(0.794,0.983)	2.233(1.540,3.240)	1.152(0.975,1.360)	0.983(0.932,1.037)
BMI (kg/m^2)				
＜18.5	1.134(1.021,1.259)	0.763(0.522,1.117)	0.921(0.785,1.081)	1.191(1.137,1.248)
18.5 and ＜24	Reference			
≥24 and ＜28	1.065(0.936,1.210)	0.557(0.330,0.939)	0.983(0.807,1.199)	0.865(0.796,0.939)
≥28	1.058(0.864,1.295)	0.779(0.353,1.717)	0.655(0.472,0.908)	0.858(0.747,0.985)
Marital status				
Married	Reference			
Widowed, divorced, separated, or single	1.264(1.148,1.392)	1.238(0.93,1.649)	1.214(1.045,1.411)	1.201(1.123,1.286)
Medical illnesses				
Hypertension	1.051(0.951,1.162)	0.625(0.406,0.961)	0.918(0.789,1.068)	0.934(0.884,0.988)
Diabetes	1.140(0.901,1.442)	1.379(0.588,3.231)	0.777(0.550,1.099)	1.030(0.897,1.183)
Heart disease	1.148(0.996,1.323)	0.921(0.499,1.702)	0.900(0.732,1.106)	1.037(0.962,1.118)
Stroke, cerebrovascular disease	1.040(0.884,1.225)	0.509(0.168,1.540)	0.580(0.458,0.733)	1.392(1.284,1.510)
Dyslipidemia	0.835(0.628,1.112)	0.892(0.304,2.617)	1.030(0.705,1.505)	0.819(0.676,0.992)
Healthy lifestyle group				
Unfavourable	1.180(1.077,1.293)	1.549(1.141,2.103)	0.675(0.585,0.778)	1.188(1.133,1.247)
Favourable	Reference			
SES group				
Lower	Reference			
Higher	0.763(0.698,0.835)	0.620(0.461,0.834)	0.965(0.842,1.107)	0.929(0.884,0.975)
**BADL disability**				
Age (years)	1.071(1.066,1.076)	1.071(1.065,1.076)	0.962(0.951,0.973)	1.025(1.021,1.028)
Gender				
Male	Reference			
Female	1.108(0.996,1.231)	0.572(0.504,0.649)	1.046(0.853,1.284)	0.781(0.728,0.837)
Region				
East	Reference			
Centre	1.051(0.937,1.178)	1.150(0.999,1.323)	1.082(0.867,1.351)	1.005(0.934,1.080)
West	0.853(0.757,0.960)	1.006(0.870,1.163)	0.987(0.768,1.270)	1.207(1.119,1.301)
BMI (kg/m^2)				

＜18.5	0.975(0.871,1.091)	1.175(1.029,1.342)	0.900(0.725,1.118)	1.215(1.138,1.298)
18.5 and ＜24	Reference			
≥24 and ＜28	1.236(1.061,1.441)	0.598(0.454,0.786)	1.175(0.883,1.564)	0.883(0.789,0.988)
≥28	1.236(0.979,1.562)	0.879(0.608,1.270)	0.754(0.481,1.180)	0.777(0.643,0.940)
Marital status				
Married	Reference			
Widowed, divorced, separated, or single	1.104(0.976,1.248)	1.379(1.184,1.605)	1.069(0.842,1.356)	1.194(1.077,1.323)
Medical illnesses				
Hypertension	0.999(0.888,1.125)	0.880(0.748,1.035)	0.805(0.648,0.999)	0.940(0.870,1.017)
Diabetes	1.252(0.972,1.614)	0.926(0.584,1.468)	0.741(0.466,1.176)	1.020(0.856,1.215)
Heart disease	1.266(1.081,1.483)	1.012(0.795,1.289)	0.889(0.676,1.169)	0.942(0.851,1.041)
Stroke, cerebrovascular disease	1.332(1.118,1.588)	0.812(0.553,1.191)	0.473(0.353,0.633)	1.135(1.029,1.253)
Dyslipidemia	0.588(0.422,0.819)	1.061(0.711,1.584)	0.573(0.335,0.979)	0.699(0.552,0.886)
Healthy lifestyle group				
Unfavourable	1.244(1.121,1.380)	1.080(0.945,1.233)	0.722(0.594,0.877)	1.268(1.181,1.363)
Favourable	Reference			
SES group				
Lower	Reference			
Higher	0.973(0.880,1.076)	0.642(0.560,0.736)	0.773(0.637,0.938)	0.890(0.832,0.952)
**Mobility disability**				
Age (years)	1.060(1.055,1.065)	1.071(1.061,1.081)	0.946(0.938,0.954)	1.042(1.039,1.045)
Gender				
Male	Reference			
Female	1.266(1.158,1.385)	0.525(0.417,0.661)	0.823(0.713,0.951)	0.670(0.636,0.706)
Region				
East	Reference			
Centre	0.802(0.723,0.890)	2.013(1.540,2.633)	0.851(0.721,1.005)	0.973(0.920,1.029)
West	0.764(0.688,0.849)	1.804(1.374,2.369)	1.047(0.884,1.241)	1.001(0.945,1.060)
BMI (kg/m^2)				
＜18.5	1.187(1.072,1.313)	0.847(0.653,1.100)	0.856(0.729,1.005)	1.179(1.122,1.239)
18.5 and ＜24	Reference			
≥24 and ＜28	1.127(0.991,1.282)	0.61(0.419,0.888)	0.926(0.755,1.136)	0.843(0.772,0.921)
≥28	1.318(1.070,1.622)	0.78(0.412,1.477)	0.748(0.531,1.054)	0.824(0.711,0.956)
Marital status				
Married	Reference			
Widowed, divorced, separated, or single	1.227(1.113,1.353)	1.183(0.951,1.472)	1.166(0.995,1.365)	1.214(1.128,1.307)
Medical illnesses				
Hypertension	1.166(1.055,1.290)	0.730(0.544,0.979)	0.925(0.791,1.081)	0.915(0.862,0.971)
Diabetes	1.233(0.960,1.583)	1.127(0.527,2.412)	0.895(0.623,1.285)	1.027(0.881,1.197)
Heart disease	1.204(1.044,1.389)	0.904(0.580,1.410)	0.849(0.684,1.054)	1.006(0.928,1.091)
Stroke,	1.062(0.898,1.257)	0.889(0.506,1.563)	0.599(0.473,0.759)	1.348(1.237,1.469)

cerebrovascular disease				
Dyslipidemia	0.843(0.617,1.150)	0.925(0.408,2.099)	1.086(0.719,1.641)	0.786(0.634,0.974)
Healthy lifestyle group				
Unfavourable	1.189(1.086,1.302)	1.280(1.026,1.596)	0.688(0.598,0.792)	1.178(1.118,1.241)
Favourable	Reference			
SES group				
Lower	Reference			
Higher	0.755(0.690,0.826)	0.619(0.499,0.769)	0.822(0.715,0.946)	0.954(0.905,1.006)
**Visual disability**				
Age (years)	1.047(1.040,1.053)	1.078(1.074,1.083)	0.971(0.963,0.980)	1.04(1.035,1.045)
Gender				
Male	Reference			
Female	1.267(1.105,1.454)	0.629(0.574,0.690)	0.952(0.800,1.131)	0.784(0.717,0.858)
Region				
East	Reference			
Centre	0.962(0.829,1.116)	1.102(0.995,1.222)	0.892(0.736,1.080)	0.944(0.861,1.036)
West	0.971(0.835,1.131)	1.025(0.923,1.139)	0.903(0.743,1.098)	0.913(0.832,1.001)
BMI (kg/m^2)				
＜18.5	1.048(0.911,1.206)	1.182(1.073,1.302)	0.816(0.682,0.976)	1.155(1.064,1.255)
18.5 and ＜24	Reference			
≥24 and ＜28	0.991(0.813,1.208)	0.774(0.659,0.908)	0.936(0.727,1.204)	0.935(0.801,1.091)
≥28	1.082(0.782,1.496)	0.740(0.540,1.014)	0.907(0.606,1.356)	1.026(0.811,1.298)
Marital status				
Married	Reference			
Widowed, divorced, separated, or single	1.247(1.064,1.463)	1.272(1.139,1.420)	1.013(0.830,1.236)	1.229(1.07,1.411)
Medical illnesses				
Hypertension	0.984(0.850,1.139)	0.820(0.728,0.923)	0.936(0.779,1.124)	1.068(0.968,1.179)
Diabetes	1.260(0.900,1.764)	1.187(0.900,1.566)	0.882(0.578,1.346)	0.993(0.763,1.293)
Heart disease	1.065(0.856,1.326)	1.160(0.998,1.347)	1.190(0.922,1.538)	1.001(0.868,1.154)
Stroke, cerebrovascular disease	1.132(0.896,1.430)	1.336(1.113,1.604)	0.728(0.543,0.977)	1.460(1.271,1.677)
Dyslipidemia	0.952(0.608,1.490)	0.930(0.671,1.288)	1.049(0.633,1.741)	0.661(0.457,0.956)
Healthy lifestyle group				
Unfavourable	1.252(1.089,1.440)	1.222(1.107,1.349)	0.614(0.518,0.728)	1.174(1.061,1.298)
Favourable	Reference			
SES group				
Lower	Reference			
Higher	0.722(0.629,0.828)	0.845(0.768,0.929)	0.844(0.713,1.000)	0.910(0.829,0.998)
**Hearing disability**				
Age (years)	1.093(1.084,1.102)	1.077(1.073,1.082)	0.990(0.974,1.005)	1.024(1.019,1.029)
Gender				
Male	Reference			
Female	0.925(0.812,1.055)	0.624(0.567,0.686)	0.864(0.715,1.045)	0.863(0.798,0.933)

Region				
East	Reference			
Centre	1.080(0.934,1.249)	1.198(1.077,1.334)	0.891(0.725,1.096)	0.857(0.791,0.929)
West	0.917(0.794,1.058)	1.067(0.959,1.188)	0.729(0.590,0.901)	0.898(0.828,0.975)
BMI (kg/m^2)				
＜18.5	1.141(0.994,1.309)	1.260(1.140,1.392)	1.157(0.957,1.397)	1.144(1.065,1.228)
18.5 and ＜24	Reference			
≥24 and ＜28	0.972(0.803,1.175)	0.821(0.704,0.956)	1.071(0.805,1.425)	0.921(0.805,1.053)
≥28	1.150(0.842,1.570)	0.842(0.632,1.122)	0.924(0.549,1.557)	0.950(0.764,1.183)
Marital status				
Married	Reference			
Widowed, divorced, separated, or single	1.415(1.214,1.649)	1.169(1.049,1.302)	0.992(0.794,1.239)	1.259(1.111,1.427)
Medical illnesses				
Hypertension	0.939(0.812,1.087)	0.918(0.824,1.024)	1.028(0.838,1.260)	0.985(0.901,1.078)
Diabetes	1.145(0.794,1.649)	1.231(0.968,1.566)	1.065(0.638,1.776)	1.004(0.779,1.295)
Heart disease	0.978(0.790,1.211)	1.127(0.975,1.303)	1.022(0.757,1.379)	1.048(0.927,1.186)
Stroke, cerebrovascular disease	1.353(1.062,1.723)	1.269(1.052,1.530)	0.988(0.714,1.367)	1.414(1.247,1.603)
Dyslipidemia	0.792(0.491,1.276)	0.780(0.562,1.083)	0.912(0.426,1.952)	0.914(0.665,1.258)
Healthy lifestyle group				
Unfavourable	1.609(1.411,1.836)	1.178(1.068,1.299)	0.952(0.798,1.134)	1.212(1.116,1.315)
Favourable	Reference			
SES group				
Lower	Reference			
Higher	0.821(0.723,0.933)	0.784(0.713,0.863)	0.857(0.717,1.025)	0.967(0.894,1.047)
**Cognitive disability**				
Age (years)	1.086(1.079,1.093)	1.076(1.071,1.081)	0.979(0.967,0.991)	1.023(1.019,1.027)
Gender				
Male	Reference			
Female	1.192(1.053,1.349)	0.548(0.491,0.613)	0.798(0.661,0.963)	0.779(0.723,0.838)
Region				
East	Reference			
Centre	1.039(0.911,1.186)	1.154(1.027,1.297)	0.724(0.589,0.890)	0.906(0.842,0.975)
West	1.047(0.916,1.197)	1.002(0.886,1.133)	0.762(0.622,0.933)	0.921(0.855,0.991)
BMI (kg/m^2)				
＜18.5	1.211(1.067,1.375)	1.160(1.032,1.303)	1.134(0.944,1.361)	1.149(1.076,1.226)
18.5 and ＜24	Reference			
≥24 and ＜28	0.865(0.722,1.036)	0.817(0.693,0.963)	0.955(0.728,1.253)	0.899(0.795,1.017)
≥28	0.817(0.623,1.071)	0.758(0.552,1.041)	0.351(0.197,0.627)	0.951(0.79,1.1440)
Marital status				
Married	Reference			
Widowed, divorced, separated, or single	1.358(1.176,1.569)	1.256(1.114,1.416)	1.065(0.858,1.321)	1.110(0.991,1.242)

Medical illnesses				
Hypertension	0.940(0.824,1.071)	0.887(0.784,1.004)	0.769(0.625,0.946)	0.993(0.916,1.076)
Diabetes	0.655(0.461,0.930)	1.482(1.192,1.842)	0.594(0.324,1.089)	0.946(0.753,1.188)
Heart disease	1.102(0.912,1.332)	1.084(0.914,1.286)	1.035(0.767,1.396)	1.074(0.962,1.200)
Stroke, cerebrovascular disease	1.280(1.054,1.553)	1.149(0.928,1.422)	0.439(0.314,0.613)	1.310(1.176,1.461)
Dyslipidemia	0.618(0.400,0.956)	0.880(0.633,1.225)	0.771(0.362,1.641)	0.930(0.688,1.257)
Healthy lifestyle group				
Unfavourable	1.579(1.397,1.784)	1.110(0.996,1.238)	0.687(0.578,0.816)	1.138(1.054,1.229)
Favourable	Reference			
SES group				
Lower	Reference			
Higher	0.851(0.755,0.958)	0.727(0.653,0.809)	0.975(0.816,1.164)	1.052(0.979,1.131)

BADL: basic activity of daily living; BMI: body mass index; SES: socioeconomic status.

Table S9. Total life expectancy and years lived without five disabilities at age 65 by lifestyle level

	Total, years (95% CI)		Free of five disabilities, years (95% CI)		
	LE at age 65 year	Difference	LE at age 65 year	Difference	Percentage of total, %
**BADL disability**					
All participants (n=15121)*					
Unfavourable group	11.959(11.371,12.504)	Reference	10.472(9.944,10.999)	Reference	87.57(86.84,88.29)
Favourable group	16.897(16.294,17.460)	4.938(4.112,5.764)	14.861(14.317,15.362)	4.389(3.679,5.146)	87.94(87.19,88.70)
Males (n=6492)†					
Unfavourable group	14.677(13.825,15.529)	Reference	13.274(12.484,14.064)	Reference	90.44(89.44,91.44)
Favourable group	11.691(10.814,12.568)	2.986(1.760,4.212)	10.506(9.744,11.249)	2.768(1.636,3.900)	89.86(88.82,90.91)
Females (n=8629)†					
Unfavourable group	12.736(11.851,13.573)	Reference	10.748(9.961,11.550)	Reference	84.39(83.34,85.44)
Favourable group	18.227(17.441,18.963)	5.491(4.325,6.657)	15.788(15.074,16.456)	5.040(3.976,6.104)	86.62(85.57,87.67)
**Mobility disability**					
All participants (n=15121)*					
Unfavourable group	12.680(11.990,13.302)	Reference	8.667(8.131,9.181)	Reference	68.35(67.33,69.38)
Favourable group	17.056(16.450,17.652)	4.376(3.474,5.278)	12.088(11.598,12.559)	3.421(2.705,4.137)	70.87(69.82,71.92)
Males (n=6492)†					
Unfavourable group	11.742(10.801,12.578)	Reference	8.96(8.21,9.685)	Reference	76.32(74.87,77.76)
Favourable group	14.307(13.172,15.371)	2.565(1.127,4.003)	11.358(10.464,12.197)	2.398(1.233,3.563)	79.38(77.97,80.79)
Females (n=8629)†					
Unfavourable group	14.886(14.121,15.552)	Reference	8.936(8.362,9.535)	Reference	60.03(58.62,61.44)
Favourable group	18.747(17.986,19.487)	3.861(2.816,4.906)	12.481(11.871,13.088)	3.545(2.702,4.388)	66.58(65.12,68.03)
**Visual disability**					
All participants (n=15121)*					

Unfavourable group	12.307(11.763,12.816)	Reference	10.290(9.791,10.761)	Reference	83.61(82.8,84.43)
Favourable group	17.175(16.629,17.730)	4.868(4.088,5.770)	14.951(14.439,15.423)	4.661(3.954,5.368)	87.05(86.28,87.83)
Males (n=6492)†					
Unfavourable group	11.732(10.994,12.438)	Reference	10.230(9.550,10.893)	Reference	87.21(86.07,88.34)
Favourable group	14.974(14.060,15.888)	3.242(2.065,4.680)	13.521(12.683,14.327)	3.291(2.221,4.456)	90.30(89.27,91.33)
Females (n=8629)†					
Unfavourable group	12.997(12.196,13.756)	Reference	10.378(9.656,11.073)	Reference	79.86(78.70,81.01)
Favourable group	18.552(17.836,19.286)	5.555(4.471,6.600)	15.83(15.162,16.442)	5.452(4.474,6.295)	85.33(84.23,86.42)
**Hearing disability**					
All participants (n=15121)*					
Unfavourable group	12.547(11.940,13.125)	Reference	11.214(10.691,11.748)	Reference	89.38(88.70,90.05)
Favourable group	17.238(16.634,17.822)	4.691(3.865,5.593)	15.798(15.264,16.330)	4.584(3.837,5.342)	91.65(91.01,92.29)
Males (n=6492)†					
Unfavourable group	11.866(11.012,12.708)	Reference	10.913(10.158,11.698)	Reference	91.97(91.04,92.89)
Favourable group	14.930(13.964,15.851)	3.064(1.819,4.502)	13.990(13.148,14.856)	3.077(1.941,4.242)	93.70(92.86,94.55)
Females (n=8629)†					
Unfavourable group	13.484(12.584,14.293)	Reference	11.797(11.009,12.556)	Reference	87.49(86.53,88.44)
Favourable group	18.646(17.856,19.379)	5.162(4.012,6.207)	16.882(16.188,17.551)	5.085(4.0416,5.928)	90.54(89.63,91.44)
**Cognitive disability**					
All participants (n=15121)*					
Unfavourable group	12.714(12.101,13.281)	Reference	11.115(10.589,11.644)	Reference	87.42(86.69,88.15)
Favourable group	17.128(16.497,17.713)	4.414(3.576,5.252)	15.731(15.184,16.269)	4.616(3.858,5.374)	91.85(91.22,92.48)
Males (n=6492)†					
Unfavourable group	12.021(11.212,12.793)	Reference	10.913(10.189,11.663)	Reference	90.79(89.81,91.78)
Favourable group	14.808(13.850,15.749)	2.787(1.575,3.999)	14.085(13.216,14.982)	3.172(2.038,4.306)	95.12(94.37,95.87)
Females (n=8629)†					

Unfavourable group	13.563(12.639,14.408)	Reference	11.354(10.576,12.124)	Reference	83.71(82.65,84.78)
Favourable group	18.556(17.739,19.311)	4.993(3.803,6.183)	16.728(16.005,17.404)	5.374(4.314,6.434)	90.15(89.23,91.07)

*All LEs have been calculated with hazard ratios adjusted for age, gender, region, BMI, marital status, hypertension, diabetes, heart disease, stroke/cerebrovascular disease, dyslipidemia, and SES.

†All LEs have been calculated with hazard ratios adjusted for age, region, BMI, marital status, hypertension, diabetes, heart disease, stroke/cerebrovascular disease, dyslipidemia, and SES.

LE: life expectancy; CI: confidence intervals.

Table S10. Total life expectancy and years lived with and without disability at age 65 (95%CI) by two levels of healthy lifestyle among 13138 participants at baseline in 2008

States at age 65	LE without disability	LE with disability	Total LE	% LE without disability
**Five disabilities**				
**All participants***	9.856(9.470,10.285)	6.417(6.077,6.762)	16.274(15.710,16.828)	60.56%
Favourable group	10.045(9.566,10.523)	6.972(6.526,7.408)	17.018(16.393,17.629)	59.03%
Unfavourable group	7.267(6.728,7.784)	5.469(5.000,5.898)	12.736(11.994,13.377)	57.06%
**Males†**	8.513(7.946,9.045)	4.253(3.848,4.658)	12.766(11.979,13.465)	66.68%
Favourable group	9.716(8.867,10.549)	4.610(3.975,5.237)	14.326(13.123,15.406)	67.82%
Unfavourable group	7.623(6.844,8.338)	3.990(3.433,4.530)	11.613(10.533,12.524)	65.64%
**Females†**	8.869(8.392,9.333)	7.712(7.274,8.128)	16.581(15.985,17.139)	53.49%
Favourable group	10.221(9.632,10.802)	8.444(7.834,9.044)	18.665(17.876,19.425)	54.76%
Unfavourable group	6.493(5.706,7.280)	7.140(6.303,7.846)	13.632(12.225,14.638)	47.63%
**Lower SES‡**	8.536(8.140,8.917)	6.215(5.890,6.546)	14.752(14.238,15.222)	57.87%
Favourable group	9.987(9.436,10.507)	7.186(6.664,7.694)	17.173(16.441,17.847)	58.16%
Unfavourable group	6.688(6.120,7.257)	5.632(5.141,6.101)	12.320(11.548,12.976)	54.29%
**Higher SES‡**	10.891(10.532,11.217)	6.562(6.246,6.879)	17.452(16.966,17.886)	62.40%
Favourable group	11.798(11.360,12.183)	7.040(6.637,7.446)	18.839(18.250,19.391)	62.63%
Unfavourable group	9.192(8.538,9.768)	5.743(5.227,6.272)	14.935(14.069,15.692)	61.55%
**BADL disability**				
**All participants***	13.745(13.256,14.236)	1.831(1.684,1.984)	15.576(15.038,16.118)	88.24%
Favourable group	14.695(14.128,15.215)	2.080(1.879,2.293)	16.775(16.144,17.363)	87.60%
Unfavourable group	10.356(9.793,10.901)	1.464(1.298,1.633)	11.820(11.199,12.393)	87.61%
**Males†**	11.562(10.99,12.128)	1.225(1.067,1.392)	12.787(12.146,13.383)	90.42%
Favourable group	13.17(12.268,14.041)	1.397(1.135,1.678)	14.568(13.562,15.521)	90.40%
Unfavourable group	10.252(9.459,11.008)	1.102(0.902,1.317)	11.355(10.506,12.138)	90.29%
**Females†**	13.571(13.022,14.111)	2.207(2.025,2.398)	15.778(15.140,16.339)	86.01%
Favourable group	15.499(14.765,16.204)	2.537(2.250,2.850)	18.036(17.219,18.805)	85.93%
Unfavourable group	10.652(9.831,11.502)	1.983(1.721,2.256)	12.636(11.721,13.503)	84.30%
**Lower SES‡**	12.100(11.654,12.528)	1.807(1.661,1.953)	13.907(13.405,14.386)	87.01%
Favourable group	14.496(13.831,15.098)	2.147(1.892,2.400)	16.643(15.926,17.323)	87.10%
Unfavourable group	9.755(9.141,10.329)	1.557(1.363,1.745)	11.312(10.659,11.929)	86.24%
**Higher SES‡**	14.952(14.530,15.339)	2.405(2.246,2.570)	17.357(16.892,17.788)	86.14%
Favourable group	16.021(15.498,16.503)	2.698(2.474,2.924)	18.719(18.122,19.251)	85.59%
Unfavourable group	12.863(12.122,13.545)	1.989(1.757,2.234)	14.852(14.057,15.593)	86.61%
**Mobility disability**				
**All participants***	11.711(11.266,12.169)	4.522(4.243,4.806)	16.233(15.669,16.793)	72.14%
Favourable group	12.044(11.536,12.540)	4.906(4.554,5.266)	16.952(16.322,17.568)	71.05%
Unfavourable group	8.681(8.130,9.215)	4.141(3.791,4.494)	12.822(12.142,13.429)	67.70%
**Males†**	10.045(9.454,10.612)	2.739(2.448,3.046)	12.785(12.011,13.476)	78.57%
Favourable group	11.305(10.37,12.188)	2.892(2.444,3.360)	14.197(13.033,15.283)	79.63%
Unfavourable group	8.951(8.155,9.723)	2.666(2.260,3.076)	11.617(10.594,12.500)	77.05%

**Females**†	10.865(10.365,11.351)	5.816(5.464,6.164)	16.681(16.084,17.236)	65.13%
Favourable group	12.421(11.786,13.051)	6.256(5.755,6.759)	18.676(17.885,19.446)	66.51%
Unfavourable group	8.242(7.424,9.051)	5.554(4.937,6.110)	13.796(12.710,14.703)	59.74%
**Lower SES**‡	10.167(9.737,10.573)	4.563(4.298,4.836)	14.731(14.219,15.196)	69.02%
Favourable group	11.933(11.320,12.477)	5.155(4.726,5.577)	17.088(16.344,17.764)	69.83%
Unfavourable group	8.157(7.542,8.758)	4.184(3.803,4.564)	12.342(11.614,13.005)	66.09%
**Higher SES**‡	12.779(12.390,13.147)	4.717(4.456,4.981)	17.496(17.014,17.928)	73.04%
Favourable group	13.850(13.357,14.296)	5.041(4.705,5.379)	18.891(18.292,19.424)	73.32%
Unfavourable group	10.847(10.127,11.500)	4.227(3.821,4.633)	15.074(14.231,15.78)	71.96%
**Visual disability**				
**All participants***	13.896(13.458,14.344)	2.046(1.890,2.214)	15.943(15.453,16.457)	87.16%
Favourable group	14.708(14.180,15.203)	2.302(2.080,2.531)	17.010(16.435,17.593)	86.47%
Unfavourable group	9.811(9.309,10.287)	2.009(1.794,2.241)	11.820(11.251,12.351)	83.00%
**Males**†	11.322(10.785,11.828)	1.543(1.343,1.753)	12.865(12.28,13.429)	88.01%
Favourable group	13.194(12.301,14.032)	1.542(1.234,1.890)	14.735(13.789,15.673)	89.54%
Unfavourable group	9.973(9.248,10.652)	1.492(1.235,1.776)	11.465(10.709,12.185)	86.99%
**Females**†	13.399(12.908,13.868)	2.726(2.504,2.945)	16.125(15.579,16.652)	83.09%
Favourable group	15.735(15.042,16.379)	2.772(2.475,3.077)	18.507(17.760,19.274)	85.02%
Unfavourable group	10.187(9.428,10.913)	2.559(2.221,2.914)	12.746(11.906,13.567)	79.92%
**Lower SES**‡	11.870(11.439,12.281)	2.061(1.888,2.231)	13.931(13.440,14.382)	85.21%
Favourable group	9.735(9.323,10.115)	2.042(1.834,2.229)	11.777(11.297,12.231)	82.66%
Unfavourable group	9.334(8.747,9.892)	1.982(1.731,2.233)	11.317(10.668,11.922)	82.48%
**Higher SES**‡	15.159(14.746,15.541)	2.256(2.089,2.415)	17.415(16.945,17.847)	87.05%
Favourable group	16.642(16.107,17.122)	2.265(2.060,2.461)	18.908(18.314,19.455)	88.02%
Unfavourable group	12.510(11.822,13.143)	2.206(1.922,2.486)	14.715(13.934,15.430)	85.02%
**Hearing disability**				
**All participants***	14.700(14.221,15.174)	1.371(1.243,1.511)	16.071(15.554,16.612)	91.47%
Favourable group	15.652(15.102,16.204)	1.493(1.327,1.674)	17.145(16.522,17.755)	91.29%
Unfavourable group	11.024(10.474,11.578)	1.337(1.175,1.511)	12.361(11.728,12.949)	89.18%
**Males**†	12.060(11.487,12.628)	1.002(0.861,1.162)	13.062(12.411,13.681)	92.33%
Favourable group	13.819(12.944,14.719)	0.976(0.762,1.217)	14.795(13.781,15.739)	93.40%
Unfavourable group	10.697(9.917,11.504)	0.976(0.787,1.206)	11.673(10.793,12.512)	91.64%
**Females**†	14.681(14.136,15.193)	1.724(1.559,1.894)	16.405(15.796,16.967)	89.49%
Favourable group	16.707(15.972,17.401)	1.841(1.601,2.101)	18.548(17.725,19.303)	90.07%
Unfavourable group	11.654(10.874,12.433)	1.721(1.459,2.000)	13.375(12.449,14.220)	87.13%
**Lower SES**‡	12.916(12.446,13.344)	1.418(1.278,1.558)	14.333(13.825,14.791)	90.11%
Favourable group	15.559(14.872,16.178)	1.537(1.321,1.758)	17.096(16.360,17.765)	91.01%
Unfavourable group	10.389(9.766,10.984)	1.368(1.183,1.562)	11.757(11.067,12.376)	88.36%
**Higher SES**‡	16.137(15.702,16.528)	1.479(1.345,1.611)	17.616(17.154,18.050)	91.60%
Favourable group	17.686(17.135,18.176)	1.442(1.278,1.607)	19.128(18.520,19.663)	92.46%
Unfavourable group	13.401(12.636,14.061)	1.513(1.292,1.750)	14.914(14.095,15.660)	89.86%
**Cognitive disability**				
**All participants***	14.593(14.100,15.088)	1.329(1.208,1.465)	15.923(15.387,16.477)	91.65%
Favourable group	15.570(14.998,16.134)	1.392(1.236,1.560)	16.962(16.306,17.582)	91.79%

Unfavourable group	10.905(10.361,11.453)	1.606(1.425,1.798)	12.511(11.876,13.095)	87.16%
**Males†**	12.083(11.498,12.653)	0.928(0.792,1.077)	13.010(12.368,13.620)	92.87%
Favourable group	13.931(13.016,14.878)	0.685(0.516,0.882)	14.616(13.612,15.597)	95.31%
Unfavourable group	10.748(9.991,11.531)	1.084(0.889,1.303)	11.832(10.995,12.626)	90.84%
**Females†**	14.460(13.900,14.976)	2.043(1.863,2.229)	16.503(15.874,17.086)	87.62%
Favourable group	16.658(15.880,17.358)	1.866(1.638,2.106)	18.526(17.670,19.314)	89.92%
Unfavourable group	11.174(10.380,11.962)	2.253(1.946,2.581)	13.427(12.481,14.294)	83.22%
**Lower SES‡**	12.908(12.430,13.334)	1.587(1.445,1.729)	14.495(13.986,14.957)	89.05%
Favourable group	15.580(14.897,16.187)	1.484(1.281,1.694)	17.064(16.320,17.732)	91.30%
Unfavourable group	10.309(9.663,10.901)	1.708(1.499,1.928)	12.016(11.324,12.660)	85.79%
**Higher SES‡**	16.163(15.727,16.559)	1.506(1.379,1.631)	17.669(17.202,18.106)	91.48%
Favourable group	17.562(17.002,18.078)	1.490(1.336,1.642)	19.052(18.444,19.610)	92.18%
Unfavourable group	13.636(12.855,14.318)	1.503(1.296,1.721)	15.139(14.299,15.872)	90.07%

*Estimated life expectancy with and without disability at age 65 according to two levels of healthy lifestyle among 13138 participants. All LEs have been calculated with hazard ratios adjusted for age, gender, region, BMI, marital status, hypertension, diabetes, heart disease, stroke/cerebrovascular disease, dyslipidemia, and SES.

†Estimated life expectancy with and without disability at age 65 according to two levels of healthy lifestyle for 5651 males and 7487 females, respectively. All LEs have been calculated with hazard ratios adjusted for age, region, BMI, marital status, hypertension, diabetes, heart disease, stroke/cerebrovascular disease, dyslipidemia, and SES.

‡Estimated life expectancy with and without disability at age 65 according to two levels of healthy lifestyle for 7605 lower SES and 5533 higher SES group, respectively. All LEs have been calculated with hazard ratios adjusted for age, gender, region, BMI, marital status, hypertension, diabetes, heart disease, stroke/cerebrovascular disease, and dyslipidemia.

BADL: basic activity of daily living; SES: socioeconomic status; LE: life expectancy.

Table S11. Total life expectancy and years lived with and without disability at age 65 by two levels of healthy lifestyle among 11853 participants without any of the five disabilities at baseline

States at age 65	LE without disability	LE with disability	Total LE	% LE without disability
**Five disabilities**				
**All participants***	10.643(10.183,11.122)	6.562(6.114,7.040)	17.205(16.512,17.900)	61.86%
Favourable group	10.817(10.265,11.419)	7.012(6.461,7.594)	17.829(17.059,18.632)	60.67%
Unfavourable group	8.066(7.444,8.770)	5.601(4.982,6.249)	13.667(12.708,14.697)	59.02%
**Males†**	9.455(8.827,10.193)	4.231(3.759,4.719)	13.686(12.821,14.606)	69.09%
Favourable group	10.571(9.603,11.652)	4.242(3.576,4.926)	14.813(13.516,16.134)	71.36%
Unfavourable group	8.447(7.615,9.373)	4.214(3.551,4.919)	12.661(11.463,13.931)	66.72%
**Females†**	10.023(9.462,10.620)	8.459(7.803,9.129)	18.483(17.614,19.362)	54.23%
Favourable group	10.996(10.285,11.733)	8.982(8.133,9.851)	19.978(18.864,21.080)	55.04%
Unfavourable group	7.657(6.613,8.851)	7.331(6.021,8.552)	14.988(13.241,16.781)	51.09%
**Lower SES‡**	9.758(9.293,10.227)	6.612(6.151,7.107)	16.37(15.677,17.078)	59.61%
Favourable group	11.510(10.896,12.130)	6.787(6.121,7.478)	18.297(17.356,19.231)	62.91%
Unfavourable group	8.084(7.263,8.868)	5.659(4.941,6.438)	13.743(12.632,14.875)	58.82%
**Higher SES‡**	11.625(11.248,12.015)	6.246(5.882,6.658)	17.871(17.282,18.456)	65.05%
Favourable group	12.567(12.097,13.057)	6.516(6.060,7.026)	19.083(18.350,19.780)	65.85%
Unfavourable group	9.281(8.645,9.948)	6.031(5.405,6.738)	15.312(14.295,16.327)	60.61%
**BADL disability**				
**All participants***	14.019(13.546,14.500)	1.770(1.628,1.931)	15.790(15.258,16.322)	88.78%
Favourable group	15.121(14.573,15.730)	1.978(1.780,2.178)	17.099(16.498,17.760)	88.43%
Unfavourable group	10.934(10.332,11.597)	1.512(1.337,1.692)	12.446(11.807,13.161)	87.85%
**Males†**	11.934(11.356,12.583)	1.225(1.063,1.394)	13.158(12.517,13.871)	90.70%
Favourable group	13.361(12.475,14.328)	1.397(1.126,1.677)	14.758(13.792,15.809)	90.53%
Unfavourable group	10.729(9.968,11.528)	1.127(0.910,1.351)	11.856(10.996,12.731)	90.49%
**Females†**	14.311(13.742,14.940)	2.148(1.953,2.335)	16.460(15.845,17.138)	86.94%
Favourable group	16.141(15.427,16.926)	2.389(2.108,2.675)	18.530(17.738,19.370)	87.11%
Unfavourable group	11.505(10.570,12.573)	1.921(1.630,2.215)	13.426(12.394,14.541)	85.69%
**Lower SES‡**	12.799(12.316,13.297)	1.743(1.598,1.898)	14.543(14.012,15.074)	88.01%
Favourable group	14.880(14.254,15.491)	2.062(1.836,2.320)	16.942(16.235,17.657)	87.83%
Unfavourable group	10.067(9.378,11.055)	1.543(1.332,1.787)	11.610(10.902,12.534)	86.71%
**Higher SES‡**	15.408(15.008,15.819)	2.261(2.100,2.441)	17.669(17.204,18.140)	87.20%
Favourable group	16.721(16.221,17.229)	2.231(2.019,2.476)	18.952(18.360,19.557)	88.23%
Unfavourable group	13.355(12.639,14.061)	1.902(1.656,2.171)	15.258(14.440,16.067)	87.53%
**Mobility disability**				
**All participants***	12.038(11.572,12.527)	4.521(4.195,4.870)	16.559(15.948,17.161)	72.70%
Favourable group	12.395(11.852,13.000)	4.981(4.572,5.415)	17.375(16.706,18.125)	71.34%
Unfavourable group	9.384(8.746,10.100)	3.907(3.478,4.345)	13.292(12.482,14.182)	70.60%
**Males†**	10.697(10.060,11.430)	2.720(2.399,3.067)	13.417(12.607,14.279)	79.73%
Favourable group	11.536(10.587,12.600)	2.846(2.376,3.349)	14.382(13.222,15.624)	80.21%

Unfavourable group	9.740(8.878,10.676)	2.666(2.222,3.122)	12.406(11.315,13.567)	78.51%
**Females†**	11.766(11.200,12.365)	6.040(5.583,6.521)	17.806(17.085,18.581)	66.08%
Favourable group	12.978(12.278,13.737)	6.360(5.728,7.009)	19.338(18.399,20.314)	67.11%
Unfavourable group	9.301(8.288,10.421)	5.587(4.785,6.405)	14.888(13.551,16.283)	62.47%
**Lower SES‡**	11.231(10.76,11.703)	4.624(4.290,4.985)	15.855(15.250,16.466)	70.84%
Favourable group	13.063(12.455,13.674)	4.550(4.060,5.085)	17.613(16.770,18.447)	74.17%
Unfavourable group	9.673(8.909,10.491)	4.095(3.617,4.615)	13.768(12.791,14.760)	70.26%
**Higher SES‡**	13.272(12.868,13.679)	4.421(4.140,4.750)	17.693(17.155,18.222)	75.01%
Favourable group	14.308(13.802,14.808)	4.591(4.233,5.002)	18.899(18.234,19.550)	75.71%
Unfavourable group	11.122(10.436,11.828)	4.049(3.580,4.586)	15.172(14.264,16.097)	73.31%
**Visual disability**				
**All participants***	14.458(13.976,14.978)	1.891(1.743,2.045)	16.349(15.834,16.863)	88.43%
Favourable group	15.044(14.468,15.640)	2.115(1.907,2.324)	17.159(16.514,17.799)	87.67%
Unfavourable group	10.749(10.148,11.399)	1.891(1.685,2.105)	12.640(11.987,13.357)	85.04%
**Males†**	11.732(11.170,12.342)	1.452(1.264,1.650)	13.182(12.572,13.854)	89.00%
Favourable group	13.378(12.452,14.386)	1.374(1.090,1.678)	14.750(13.704,15.820)	90.70%
Unfavourable group	10.502(9.652,11.320)	1.392(1.118,1.662)	11.894(11.004,12.726)	88.30%
**Females†**	13.988(13.438,14.572)	2.574(2.360,2.794)	16.562(15.944,17.218)	84.46%
Favourable group	16.040(15.260,16.818)	2.596(2.308,2.888)	18.636(17.754,19.448)	86.07%
Unfavourable group	11.152(10.230,12.172)	2.426(2.086,2.790)	13.578(12.568,14.688)	82.13%
**Lower SES‡**	12.708(12.167,13.274)	1.877(1.716,2.042)	14.584(13.981,15.184)	87.13%
Favourable group	15.077(14.324,15.782)	2.115(1.853,2.366)	17.192(16.340,17.996)	87.70%
Unfavourable group	10.222(8.809,13.530)	1.839(1.041,2.371)	12.061(10.723,14.953)	84.75%
**Higher SES‡**	15.355(14.936,15.768)	2.087(1.935,2.256)	17.442(16.966,17.932)	88.03%
Favourable group	16.771(16.215,17.314)	2.050(1.865,2.252)	18.821(18.193,19.440)	89.11%
Unfavourable group	12.828(12.154,13.492)	2.098(1.840,2.396)	14.927(14.172,15.694)	85.94%
**Hearing disability**				
**All participants***	15.399(14.930,15.881)	1.232(1.114,1.356)	16.631(16.122,17.164)	92.59%
Favourable group	16.278(15.717,16.907)	1.331(1.179,1.489)	17.609(16.991,18.259)	92.44%
Unfavourable group	11.861(11.235,12.472)	1.177(1.009,1.349)	13.038(12.364,13.683)	90.97%
**Males†**	12.610(12.025,13.204)	0.944(0.793,1.097)	13.553(12.922,14.194)	93.04%
Favourable group	14.326(13.370,15.374)	0.860(0.646,1.094)	15.186(14.182,16.270)	94.34%
Unfavourable group	11.286(10.460,12.102)	0.948(0.735,1.171)	12.233(11.271,13.141)	92.26%
**Females†**	15.155(14.565,15.733)	1.594(1.424,1.776)	16.750(16.129,17.351)	90.48%
Favourable group	17.504(16.784,18.264)	1.640(1.424,1.870)	19.144(18.354,19.960)	91.43%
Unfavourable group	12.228(11.240,13.180)	1.558(1.272,1.866)	13.784(12.730,14.762)	88.71%
**Lower SES‡**	13.771(13.258,14.262)	1.244(1.114,1.379)	15.015(14.493,15.532)	91.71%
Favourable group	16.000(15.320,16.643)	1.411(1.196,1.640)	17.411(16.701,18.136)	91.90%
Unfavourable group	11.323(10.577,12.056)	1.319(1.141,1.504)	12.642(11.848,13.402)	89.57%
**Higher SES‡**	16.304(15.878,16.714)	1.398(1.266,1.542)	17.700(17.234,18.158)	92.10%
Favourable group	17.708(17.189,18.225)	1.371(1.215,1.549)	19.079(18.500,19.660)	92.81%
Unfavourable group	14.026(13.319,14.707)	1.442(1.205,1.702)	15.469(14.699,16.253)	90.67%
**Cognitive disability**				
**All participants***	14.967(14.521,15.412)	1.314(1.188,1.452)	16.281(15.789,16.779)	91.93%

Favourable group	15.971(15.400,16.624)	1.343(1.185,1.509)	17.314(16.696,17.995)	92.24%
Unfavourable group	11.436(10.841,12.015)	1.467(1.277,1.660)	12.902(12.243,13.534)	88.64%
**Males†**	12.442(11.843,13.091)	0.902(0.768,1.045)	13.343(12.708,14.042)	93.25%
Favourable group	13.988(13.084,14.971)	0.695(0.508,0.903)	14.682(13.741,15.727)	95.27%
Unfavourable group	11.052(10.298,11.869)	1.054(0.851,1.258)	12.106(11.275,12.994)	91.29%
**Females†**	14.760(14.234,15.332)	2.016(1.806,2.226)	16.776(16.174,17.428)	87.98%
Favourable group	17.110(16.363,17.958)	1.802(1.564,2.056)	18.912(18.089,19.817)	90.47%
Unfavourable group	11.492(10.604,12.414)	2.144(1.788,2.532)	13.636(12.648,14.630)	84.28%
**Lower SES‡**	13.498(12.980,13.988)	1.492(1.336,1.652)	14.988(14.440,15.492)	90.05%
Favourable group	15.789(15.131,16.400)	1.423(1.220,1.653)	17.211(16.493,17.921)	91.74%
Unfavourable group	11.313(10.603,12.027)	1.426(1.211,1.655)	12.739(11.955,13.501)	88.81%
**Higher SES‡**	16.426(16.003,16.840)	1.459(1.336,1.597)	17.885(17.421,18.349)	91.84%
Favourable group	17.601(17.070,18.136)	1.461(1.302,1.646)	19.062(18.465,19.649)	92.34%
Unfavourable group	13.935(13.175,14.746)	1.405(1.202,1.631)	15.340(14.514,16.203)	90.84%

*Estimated life expectancy with and without disability at age 65 according to two levels of healthy lifestyle among 11853 participants. All LEs have been calculated with hazard ratios adjusted for age, gender, region, BMI, marital status, hypertension, diabetes, heart disease, stroke/cerebrovascular disease, dyslipidemia, and SES.

†Estimated life expectancy with and without disability at age 65 according to two levels of healthy lifestyle for 5550 males and 6303 females, respectively. All LEs have been calculated with hazard ratios adjusted for age, region, BMI, marital status, hypertension, diabetes, heart disease, stroke/cerebrovascular disease, dyslipidemia, and SES.

‡Estimated life expectancy with and without disability at age 65 according to two levels of healthy lifestyle for 6750 lower SES and 5103 higher SES group, respectively. All LEs have been calculated with hazard ratios adjusted for age, gender, region, BMI, marital status, hypertension, diabetes, heart disease, stroke/cerebrovascular disease, and dyslipidemia.

BADL: basic activity of daily living; SES: socioeconomic status; LE: life expectancy.

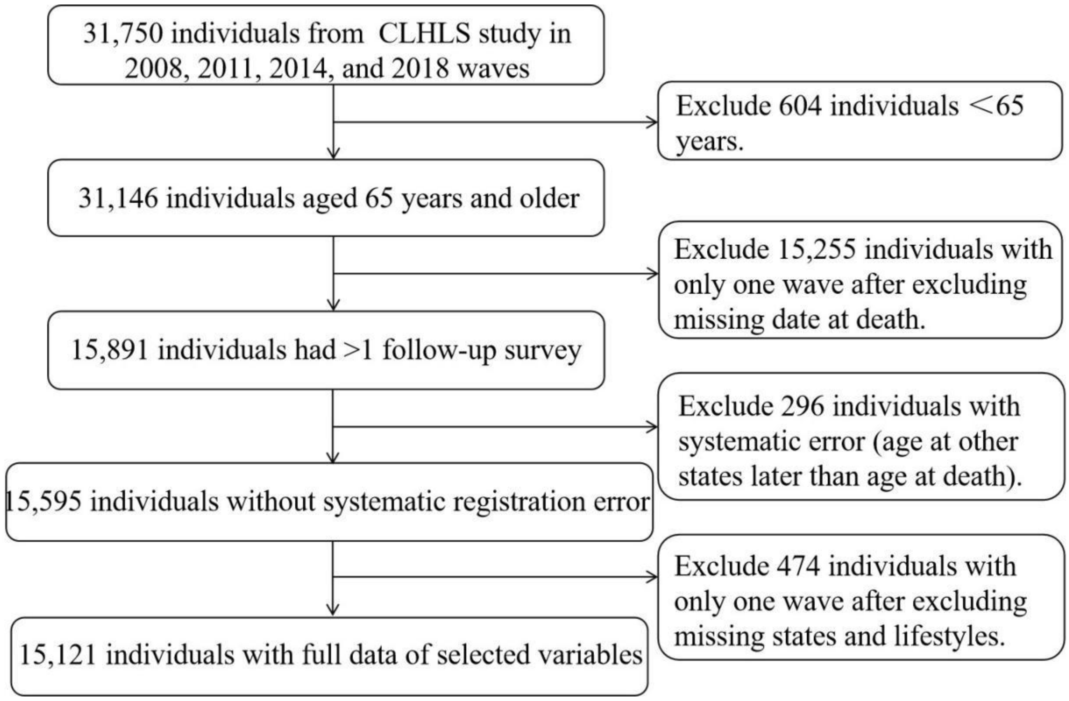

Figure S1. Flow Chart across 4 waves of the CLHLS from 2008 to 2018

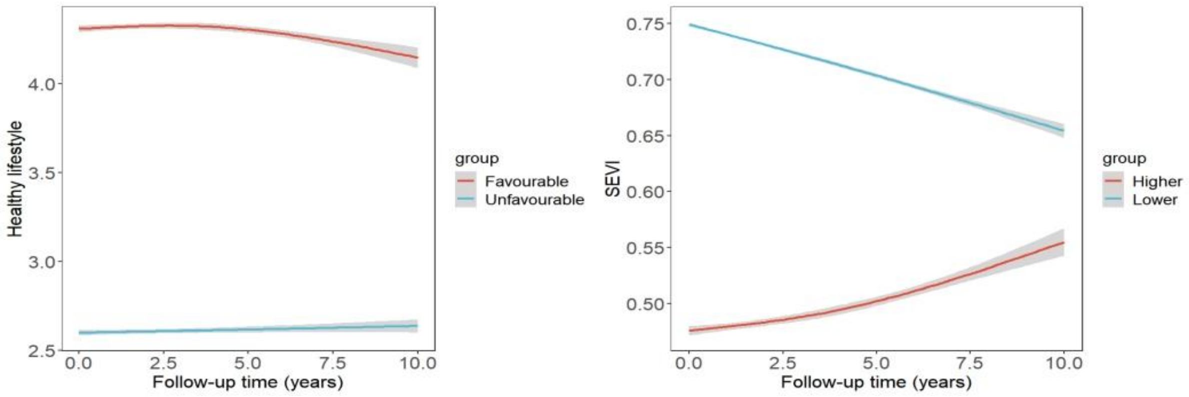

Figure S2. Trajectories of healthy lifestyle and SEVI predicted by the LCGMM among 15121 older adults. The solid line represents the average blood pressure in a class and the shaded area indicated 95% CIs

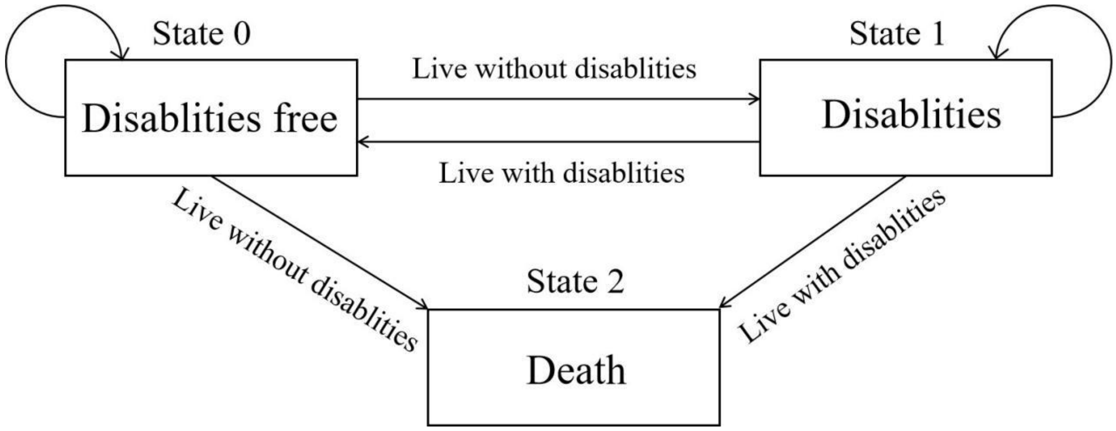

Figure S3. Markov multistate transition model over the time of follow-up applied to the multistate life table for the estimation of life expectancy

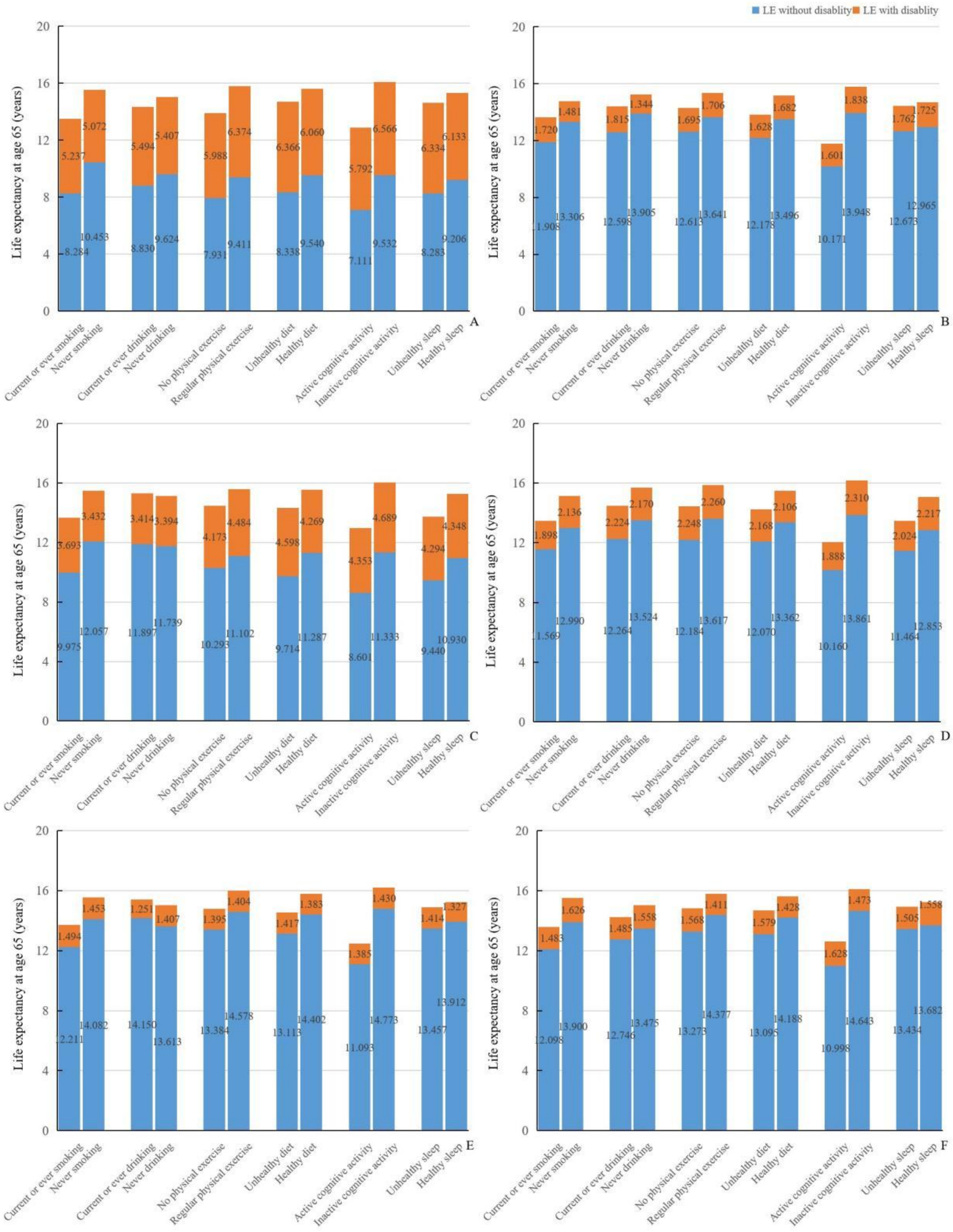

Figure S4. Estimated life expectancy (LE) with and without disability at age 65 according to levels of individual lifestyle factors among 15121 participants. All LEs have been calculated with hazard ratios adjusted for age, gender, region, BMI, marital status, hypertension, diabetes, heart disease, stroke/cerebrovascular disease, dyslipidemia, and SES. **A.** Five disabilities in combination. **B.** BADL disability. **C.** Mobility disability. **D.** Visual disability. **E.** Hearing disability. **F.** Cognitive disability.
